# Voxelized simulation of cerebral oxygen perfusion elucidates hypoxia in aged mouse cortex

**DOI:** 10.1371/journal.pcbi.1008584

**Published:** 2021-01-28

**Authors:** Grant Hartung, Shoale Badr, Mohammad Moeini, Frédéric Lesage, David Kleinfeld, Ali Alaraj, Andreas Linninger

**Affiliations:** 1 Department of Bioengineering, University of Illinois at Chicago, Chicago, Illinois, United States of America; 2 Polytechnique Montréal, Department of Electrical Engineering, Montreal, Canada; 3 Department of Physics, University of California San Diego, San Diego, California, United States of America; 4 Department of Neurosurgery, University of Illinois at Chicago, Chicago, Illinois, United States of America; Universiteit Leiden, NETHERLANDS

## Abstract

Departures of normal blood flow and metabolite distribution from the cerebral microvasculature into neuronal tissue have been implicated with age-related neurodegeneration. Mathematical models informed by spatially and temporally distributed neuroimage data are becoming instrumental for reconstructing a coherent picture of normal and pathological oxygen delivery throughout the brain. Unfortunately, current mathematical models of cerebral blood flow and oxygen exchange become excessively large in size. They further suffer from boundary effects due to incomplete or physiologically inaccurate computational domains, numerical instabilities due to enormous length scale differences, and convergence problems associated with condition number deterioration at fine mesh resolutions. Our proposed simple finite volume discretization scheme for blood and oxygen microperfusion simulations does not require expensive mesh generation leading to the critical benefit that it drastically reduces matrix size and bandwidth of the coupled oxygen transfer problem. The compact problem formulation yields rapid and stable convergence. Moreover, boundary effects can effectively be suppressed by generating very large replica of the cortical microcirculation *in silico* using an image-based cerebrovascular network synthesis algorithm, so that boundaries of the perfusion simulations are far removed from the regions of interest. Massive simulations over sizeable portions of the cortex with feature resolution down to the micron scale become tractable with even modest computer resources. The feasibility and accuracy of the novel method is demonstrated and validated with *in vivo* oxygen perfusion data in cohorts of young and aged mice. Our oxygen exchange simulations quantify steep gradients near penetrating blood vessels and point towards pathological changes that might cause neurodegeneration in aged brains. This research aims to explain mechanistic interactions between anatomical structures and how they might change in diseases or with age. Rigorous quantification of age-related changes is of significant interest because it might aide in the search for imaging biomarkers for dementia and Alzheimer’s disease.

## Author summary

Brain function critically depends on the maintenance of physiological blood supply and metabolism in the cortex. Disturbances to adequate perfusion have been linked to age-related neurodegeneration. However, the precise correlation between age-related hemodynamic changes and the resulting decline in oxygen delivery is not well understood and has not been quantified. Therefore, we introduce a new compact, and therefore highly scalable, computational method for predicting the physiological relationship between hemodynamics and cortical oxygen perfusion for large sections of the cortical microcirculation. We demonstrate the novel *mesh generation-free* (MGF), multi-scale simulation approach through realistic *in vivo* case studies of cortical microperfusion in the mouse brain. We further validate mechanistic correlations and a quantitative relationship between blood flow and brain oxygenation using experimental data from cohorts of young, middle aged and old mouse brains. Our computational approach overcomes size and performance limitations of previous unstructured meshing techniques to enable the prediction of oxygen tension with a spatial resolution of least two orders of magnitude higher than previously possible. Our simulation results support the hypothesis that structural changes in the microvasculature induce hypoxic pockets in the aged brain that are absent in the healthy, young mouse.

This is a *PLOS Computational Biology* Methods paper.

## Introduction

Late-onset neurodegenerative diseases are believed to interrupt or degrade the coordination between vascular blood flow and neural tissue oxygenation. For example, hypoxic events have been implicated in beta-amyloid production in Alzheimer’s Disease (AD) [1,2]. Patients with various forms of dementia also experience diminished oxygen tension in addition to significant morphological changes in cerebrovascular angioarchitecture [3–8]. Unfortunately, normal aging (aging without dementia) also entails similar morphological changes [3], so that normal age related changes are hard to distinguish from diseases. In order to better diagnose and eventually treat age-related dementia, it is imperative to be able to precisely quantify changes in vascular topology that potentially impair adequate oxygen supply to the brain. While the link between vascular restructuring and reduced tissue oxygenation have been established [4,9–12], the quantitative contribution of hemodynamic factors to abnormal oxygen distribution have not been characterized due to the difficulty in posing and solving problems on a massive scale.

Multimodal medical images techniques enable the acquisition of detailed measurements about the microcirculation such as hematocrit, blood flow velocities, and oxygen concentration in the vasculature or the extravascular space [1,2,4,9,11–13]. However, these invaluable data are typically collected from different specimen at different locations or time points with widely varying resolutions pertaining to a host of imaging modalities (μCT, 2PLSM, MRI). To direct the systematic search for imaging biomarkers of neurodegenerative diseases, it would be ideal to be able to focus data and observations across different length and time scales from diverse imaging modalities into a single coherent picture of hemodynamics and microperfusion in normal and pathological states. With this objective in mind, several groups have pursued computer simulations to quantify mechanistic interactions between cerebral blood flow and oxygen exchange supported by multimodal imaging data at the microlevel [14–25]. Especially, anatomically accurate models which recreate the detailed cerebral micro-circuitry bring the advantage that measurements at all length scales or acquired with separate imaging modalities can be combined *in silico* to make quantitative predictions about the relationship between blood flow and metabolism. Unfortunately, the solution of anatomically realistic mathematical models for cerebral circulation and oxygen exchange is a massive computational task.

Several computational models have been proposed to investigate cerebral oxygen metabolism [14,26–35]. They can be grouped into (i) analytic and semi-analytic, and (ii) finite element methods (FEM).

*Analytical and semi-analytical solutions of oxygen exchange*. Analytical approaches such as the Green function method [31,32] approximate tissue oxygen tension by evaluation of an infinite series for oxygen supply from point or line sources representing blood vessels into a three-dimensional tissue domain. Most analytic and semi-analytic singularity removal methods require summations of analytical expressions for concentrations and fluxes from single point or line sources into the domain, while satisfying homogenous boundary conditions. While such formulations are available for simple geometries (e.g. sphere, cube, cylinder), it is not obvious how to generate analytical formulations delineating complex biological spaces such as the highly gyrated cerebral cortex.

*Methods using unstructured finite element meshes*. Numerous groups have combined finite element discretization with 1D network graphs to create so called 1D-3D coupling approaches [26–28,30,36]. These elegant formulations will be reviewed in more detail in the discussion but a few selections are introduced here.

In a recent example, an FEM method was used to simulate extracellular species transport was in high resolution mesh measuring ~4x4x4μm^3^ with ~82–84 million tetrahedrons [37]. As mentioned, FEM body-fitted meshes require contiguous representation of the space covering vascular segments and extravascular space. Even though excellent meshing tools are available such as ANSYS ICEM (Canonsburg PA), HyperMesh (Troy MI) and GMSH [38], segmentation of the cerebral microdomain for generating body fitted meshes precisely delineating capillaries, endothelium and parenchyma is a limiting factor in our experience. This bottleneck exists because automatic meshing often fails in the tortuous and highly bifurcated capillary bed, thus requiring manual corrections which is impractical due to the large number of segments. More importantly, tetrahedral segmentation of mixed vascular and extravascular domains entails a prohibitively large number of mesh elements needed to resolve the interface. Apart from the mesh size, it should be noted that the convergence of extremely large meshes suffers from a resolution-dependent deterioration of the matrix condition number, as shown by Briggs [39] even for the 1D diffusion. In effect, finely meshed domains necessarily result in an escalating iteration count, thus leading in practice to cases that do not converge at all or stagnate at poor approximations that does not satisfy all equations (= insufficiently small residual errors).

Another approach is to use *dual mesh techniques* where a 1D vascular network is coupled to a 3D extravascular mesh. These approaches benefit from problem reduction which improves solvability and avoids solving the 3D nonlinear Navier Stokes equations to predict vascular blood flow. Gagnon and Boas solved oxygen exchange between the microvasculature embedded in brain tissue using a tetrahedral mesh with sharp interface between the blood vessels and tissue [14,28,30]. Blood flow was computed separately using a simplified Hagen-Poiseuille network model; then oxygen extraction to tissue was solved by projecting the segment oxygen tension to the refined triangular surface mesh of each vessel segment. Other groups avoided the body-fitted meshing by registering a vascular network to a tetrahedral mesh and distributing the transmembrane flux for each segment across many tetrahedral mesh elements [33,34,40–43]. Unfortunately, reported problem sizes merely encompass up to a few thousand vascular segments, because the required mesh sizes for fitting a contiguous extravascular mesh with the vascular network graph increase dramatically especially at the microscale.

A similar *dual mesh technique* has been proposed that registered the vascular network graph to a surrounding tissue domain represented by a coarse tetrahedral mesh [26,27,36]. Mass exchange was approximated as the total flux from the entire cylindrical segment to the mesh cell containing its center. This method already enjoyed the benefit that it did not require a body fitted mesh, thus enabling oxygen simulations for sizable portions of the cerebral cortex; 3x3x3mm^3^ in humans [36]; and 1x1x1mm^3^ in mouse [27]. Furthermore, the overall CPU time could be reduced by solving oxygen tension over the two meshes simultaneously, instead of having to iteratively solve oxygen fields in the blood and tissue domains separately. Unfortunately, point coupling between the centerline cylinder with surrounding tissue cell does not precisely orient and distribute the transmembrane flux across the endothelial interface, although the issue could be partially resolved using anisotropic and/or adaptive meshes.

Dual-meshes were also used to solve dynamic flow problems solving the Navier Stokes equations for fluid flowing within deformable domain boundaries [44,45]. A technique floating a moving (= Lagrangian) mesh inside a fixed (= Eulerian) mesh frame avoided remeshing in every time step, which would otherwise be necessary when simulating distensible blood vessels or a suspension of deformable red blood cells.

It is well known that parametric, structured meshing can drastically accelerate the convergence of transport problems. Several authors have developed *homogenization* methods to overcome the need for massive tetrahedral meshes by replacing body-fitted meshing with Cartesian meshes and anisotropic diffusion tensors [46,47]. Unfortunately, these methods do not capture discrete paths the blood takes through the capillary bed, so it is not yet obvious how homogenized model results can be interpreted or validated. These methods are discussed in more detail in the discussion section. Other groups performed dynamic 3D hemodynamic simulations effectively solving blood flow in the visible portion of the main cerebral arteries using parametric structured body fitted meshes [48–50]. The Sarntinoranont group introduced an elegant *voxel-based* method for predicting drug dispersion in spinal tissue; her method had the advantage that it seamlessly integrated the information flow between image data and simulations by aligning simulation results with image data at the same Cartesian “voxel” grid [51]. Here, we introduce a novel voxel based meshing-less discretization method that uses multiscale, parametric, structured grid techniques that drastically reduces problem size and accelerate convergence of oxygen transport simulations in the cerebral microcirculation.

Here we propose a method that rests on a structured Cartesian grid encompassing both the vascular and extravascular domain with a crisp separation interface obtained by a simple masking procedure. Masking of the interface only requires centerline a diameter information of the vascular network graph thus avoiding the need for body-fitted mesh generation altogether. This approach offers several critical advantages: (i) Masking operations can be executed using simple Euclidean geometry operators, eliminating the need for cell connectivity indexing (adjacency information). (ii) Memory storage for the Cartesian grid is minimal, since it requires only a few scalar parameters (domain bounding box and element counters for the main dimensions in x, y, and z-directions) which substantially reduces RAM requirements. (iii) Cortical oxygen perfusion problems discretized with our voxelized, mesh generation-less method enjoys excellent mathematical solvability. (iv) The cell masking paradigm is readily expandable to more specific anatomical labeling (smooth muscle domain, nonuniform endothelial thickness, or perivascular spaces). Finally, (v) the discrete labeling algorithm enforces that each vascular segment exchanges mass fluxes throughout its entire endothelial surface layer with the surrounding neighborhood of extravascular cells, which improves the computational fidelity of the predictions compared to point source 1D-3D coupling. This method is capable of computing oxygen gradients around vessels in the microvascular network at all relevant length scales. This new methodology converges simulations of significantly larger cortical microcirculatory networks than previously attempted. Moreover, the system is stable up to ~100M mesh elements and ~1M vascular segments on a desktop workstation. The substantial performance improvement puts us a step closer to whole-brain simulations.

## Results

### Structural Cartesian mesh properties

**Fig 1:**
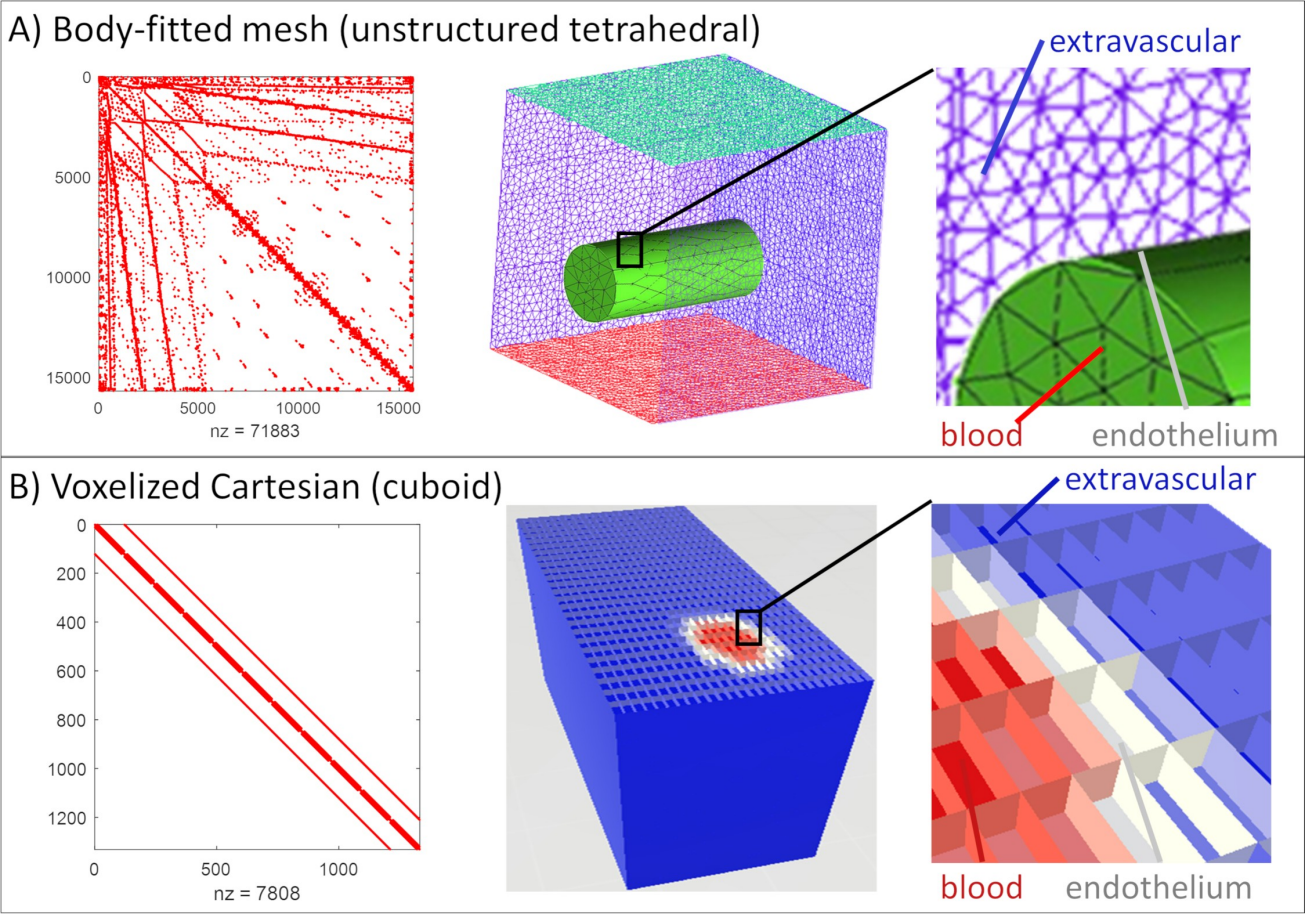
Matrix structure of diffusion problem with different meshing approaches in finite volume discretization. (A) The tetrahedral mesh matrix has a large bandwidth with no apparent block structure. In FEV discretization of unstructured domains, unknown nodal values typically occur in dozens of balance equations which augments matrix bandwidth thus substantially hampering the solvability of the underlying transport problem. (B) The structured Cartesian (cuboid) mesh covering the same domain with similar characteristic edge length has a much narrower bandwidth and has block structure which is ideally suited for block diagonalization preconditioning. Tetrahedral meshes also require almost ten times (in our example 9.2 times) more grid cells (nz) than the analogue cuboid mesh.

First we show that the proposed structured, cuboid Cartesian mesh discretization significantly improves the matrix structure of the transport equations compared to traditional tetrahedral meshing as exemplified by Fig 1. The matrix representation of finite volume or finite diffusion fluxes using tetrahedral mesh has a very wide bandwidth, because many elements (= rows) have more than several dozen entries, each one indicating exchange fluxes with other cells. On the other hand, the Cartesian mesh has a highly organized block diagonal structure with a constant element-to-element connectivity of seven. Thus, the bandwidth of the mass exchange/transport matrix stemming from the Cartesian mesh is much smaller than in the tetrahedral counterpart. Moreover, the transport coefficients in the matrix have typically the same value. Due to its superior structure, connectivity and uniformity of entries, the Cartesian mesh is more amenable to block preconditioning which enjoys more rapid and robust convergence.

Moreover, the number of elements required to represent a tissue domain with a tetrahedral mesh is significantly larger than for the Cartesian mesh. A hexahedral mesh encompassing the same space with the same characteristic edge length is, by default only ~1/8 the size of a tetrahedral mesh. Specifically, expressing the cortical section into tetrahedral elements using ANSYS ICEM [Canonsburg, PA] resulted in a ratio of tetrahedral volumes to hexahedral volumes of 9.2:1 with similar characteristic edge lengths. Accordingly, parametric structured Cartesian meshes yield a problem reduction of almost one order of magnitude.

#### *Voxelized* vascular network representation-masking

As described above, we also use the Cartesian mesh domain to precisely delineate the cortical microcirculation. This choice gives the critical advantage that it eliminate the need for automatic body-fitted mesh generation, which often fails on microcirculatory meshes or requires time consuming manual adjustments to ensure adequate mesh quality at the interface between vascular luman and extravascular space especially at the microvessel length scale of ~1*μ* m. the simple masking logic described in section 3.4 was applied to partition the “voxels” in the three dimensional domain into one of three groups; (i) interior vascular cells (red label), (ii) endothelial boundary cell (grey label), and (iii) tissue element (blue label for extravascular space). The structure of the resulting computational domain resembles a 3D image obtained from an imaging modality where the “grid resolution” determines the smallest features in the simulation. The edge length of the finest cuboid cell also delineates the minimum feature scale without the need for creating a body-fitted tetrahedral mesh. In effect, all advantages of the structural meshing described in the previous section carry over for the integrated vascular and tissue domains.

**Fig 2:**
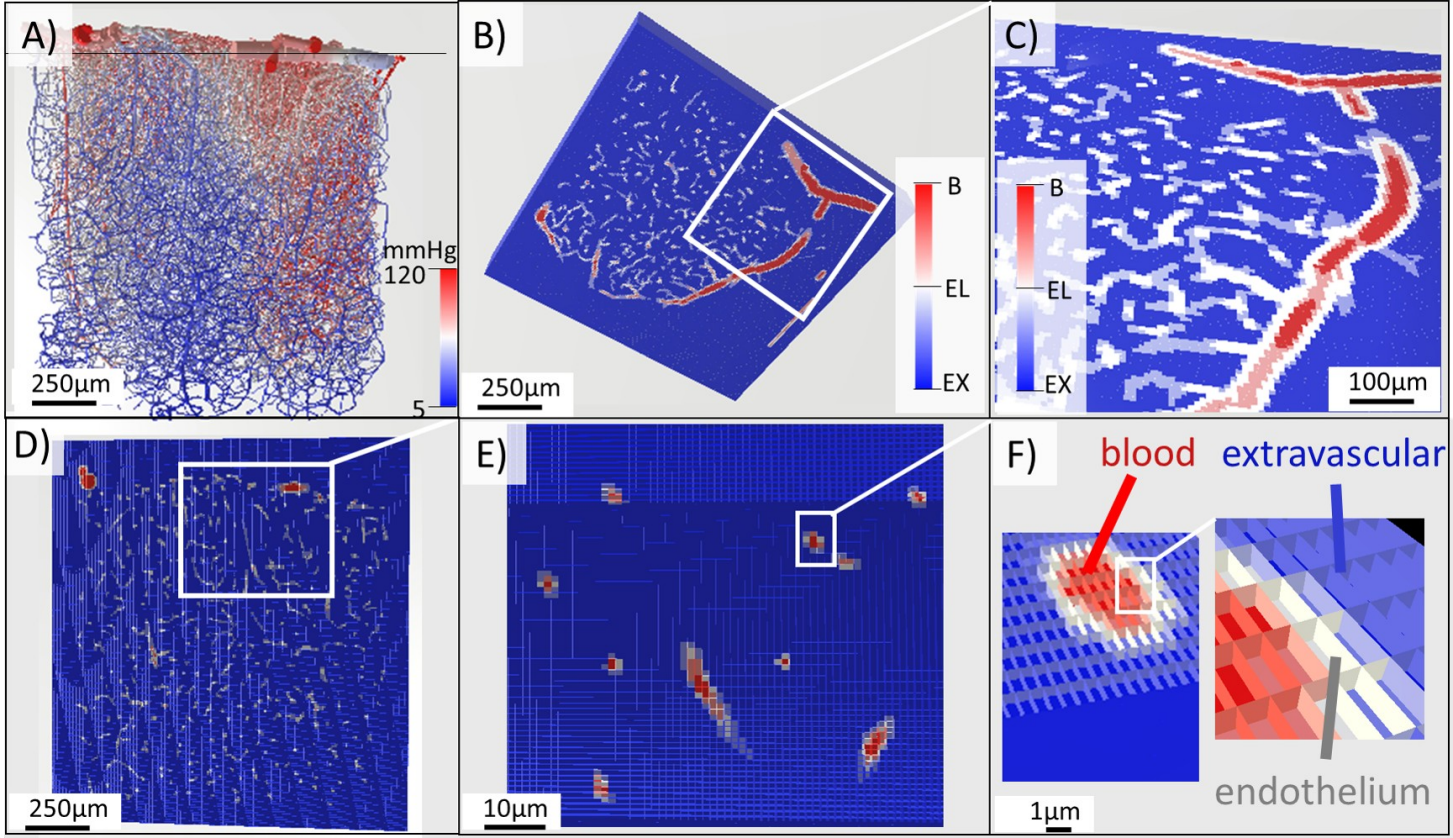
Illustration of the masking algorithm for labeling at different resolutions in the cortical microdomain. A) The cortical network (E1.1) is labeled by pressure visualizing arteries (red), veins (blue) and connecting capillaries. B-C) This cortical network has been labeled a mesh visualized at many resolutions. Coloration of mesh is tri-color; red = blood, grey = endothelial, and blue = extravascular cells. Note, most of the cells belong to the extravascular space (blue). The blood lumen is marked by intravascular elements (red). Mass transfer occurs at the interface elements (grey). D-F) Visualization of mesh labeling at three resolutions reflects the capability of the labeling to resolve the connectivity between vasculature and tissue at all length scales. F) The distribution of endothelial layer, intravascular, and extravascular elements can be seen at the finest resolution.

Results of the beneficial mesh generation of the *voxelized* vascular network are described next. The implementation of masking logic followed section 3.3. with more details in Fig 1 and S1 Text. The outcomes of the masking logic is illustrated using a microcirculatory network (~1x1x1mm^3^) of the somatosensory cortex in one of the mouse Kleinfeld dataset [27,52,53]. The labeling results for the vascular network, endothelial interface and extravascular space are depicted in Fig 2. The interior lumen of the pial arteries, penetrating arterioles, capillaries, venules and veins is labeled in red. Large caliber vessels show blocks of red elements delineated by a distinct boundary layer (white elements) as shown in Fig 2C. Thinner vessels depicted in Fig 2D and 2E may have an endothelial layer measuring only one element (= isolated grey cells). Outside the endothelial layer, blue cells constitute the extravascular tissue space (= extravascular elements). The large number of tissue cells also underscores that the bulk of the computational effort is spent on solving oxygen tension in the tissue, while the number of equations to compute flow and oxygen in blood is typically about two orders of magnitude smaller. It should also be noted that the proposed Cartesian network masking preserves the block-diagonal matrix structure discussed above as can be seen in Fig 1, Right.

### Divergence free and mesh independence simulations

We tested the ability of the Cartesian “meshing-free” method to consistently resolve microgradients around microvascular blood vessels in two large specimen of the somatosensory cortex of mouse. To this end, we tracked 25 rays through the cortical sample domain as a function of characteristic edge length. In these simulations, the convective flow field was solved a-priori using the matrix Eqs in (1) and (2). The oxygen fields in the vascular network and in the tissue mesh were converged simultaneously. For the oxygen dispersion in the vasculature, the convective flux uses the bulk blood flow and hematocrit; the mass transfer from the segment to the tissue is computed by summing the contributions of all endothelial boundary elements surrounding this segment. In effect, the oxygen field computation for the vasculature is executed on a tubular network but coupled to the surrounding tissue using the fuzzy representation of the boundary endothelial layer. With the convection equation for the vasculature and the mass transfer coupling equations for the interface and the diffusive transport and reaction equation for the tissue, the resulting vascular-tissue system in (3) is solved simultaneously using a preconditioned GMRES [54,55].

#### Divergence-free simulation

We calculated the overall mass balance error for blood flow simulation as well as for oxygen tension. Table 1 lists the results showing almost perfect closure of both bulk blood flow and oxygen balances for the test cases. We also validated divergence free simulation of the oxygen field using spot checks in all locations of the vascular and tissue domains with a maximum flow balance error of |*e*|_∞_<1.8∙10^−10^ %. Because the mass balance fluxes and reactive source terms were enforced using the finite volume approach, divergence-free computations were achieved both from the theoretical as well as the numerical perspective. More details in the finite volume discretization are given in S3 Text.

**Table 1:** Statistical properties of numerical performance of blood flow and oxygen simulations. The small loss terms demonstrate divergence free simulations.

Parameter	Value	Units
Blood flow inlet	52.9	nL/s
Blood flow outlet	52.9	nL/s
Normalized overall mass loss	2.6e-7	%
Oxygen inlet feed	258.4	nmol/s
Oxygen outlet flux	164.1	nmol/s
Oxygen mass transfer	94.3	nmol/s
Normalized oxygen overall mass loss	1.5e-8	%

#### Raytracing

**Fig 3:**
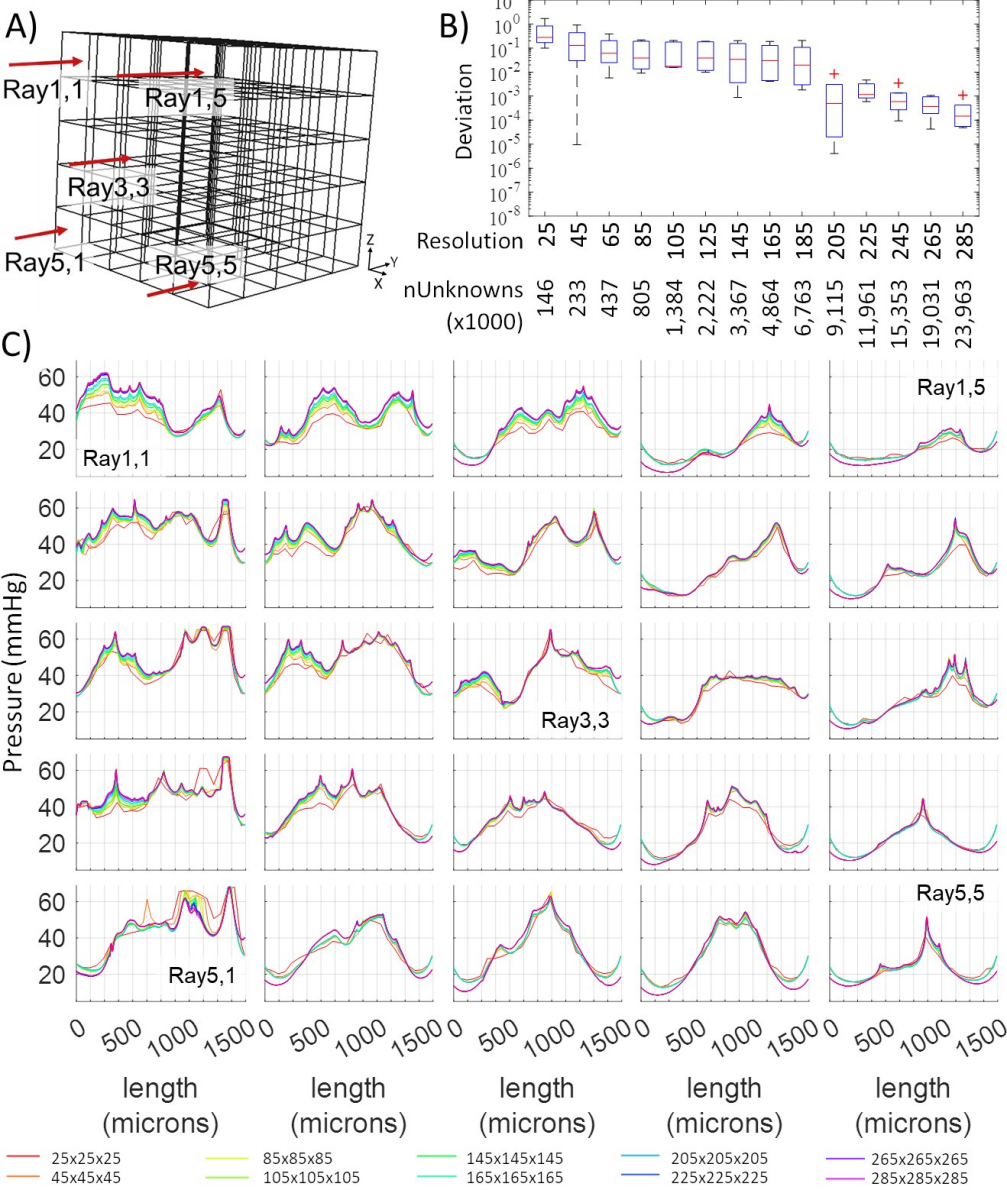
Tracing of oxygen tension across 25 rays penetrating the tissue domain (raytracing analysis) for an empirical network (specimen E4.1). A) 3D Cartesian tissue mesh and location of 25 rays to examine the variability of tissue tension as a function of grid resolution. B) Statistics of deviations of the computed oxygen profiles from the reference solution (= the finest grid with a resolution of 305x305x305 elements per volume corresponding to ~29M cells). C) Simulated oxygen profiles as a function of 15 grid resolutions along 25 rays through the domain. The oxygen simulations stabilize well below the highest resolution. Specifically, results become mesh independent at 205x205x205 domain (resolution = 205) which corresponds to ~9.115M unknowns.

Concentration profiles along rays in Fig 3 typically show sharp peaks near penetrating arterioles and valleys in locations further away and around venules. The computed oxygen concentration profiles along each ray for the first specimen (E4.1) further converged to a stable function even in the presence of steep gradients around blood vessels with high oxygen tension (= penetrating arterioles). Typically, mesh independence is achieved for meshes with 205 mesh elements per side, or a grid resolution of ~5 microns. We also show successful convergence for more refined mesh sizes down to ~3 microns, which was the limit in problem size imposed by available memory in our chosen inexpensive PC hardware (Intel Xeon W-2025, 1 core, 256GB RAM). A second large scale specimen (E1.1) exhibits similar satisfactory mesh independence with results shown in S2 Text. The oxygen simulation typically converged within 0.001–2.58 CPU hours (depending on mesh resolution). This investigation using two large scale cortical microdomains demonstrate excellent stability and mesh independence.

### Precise quantification of oxygen microgradients in the cerebral cortex

**Fig 4:**
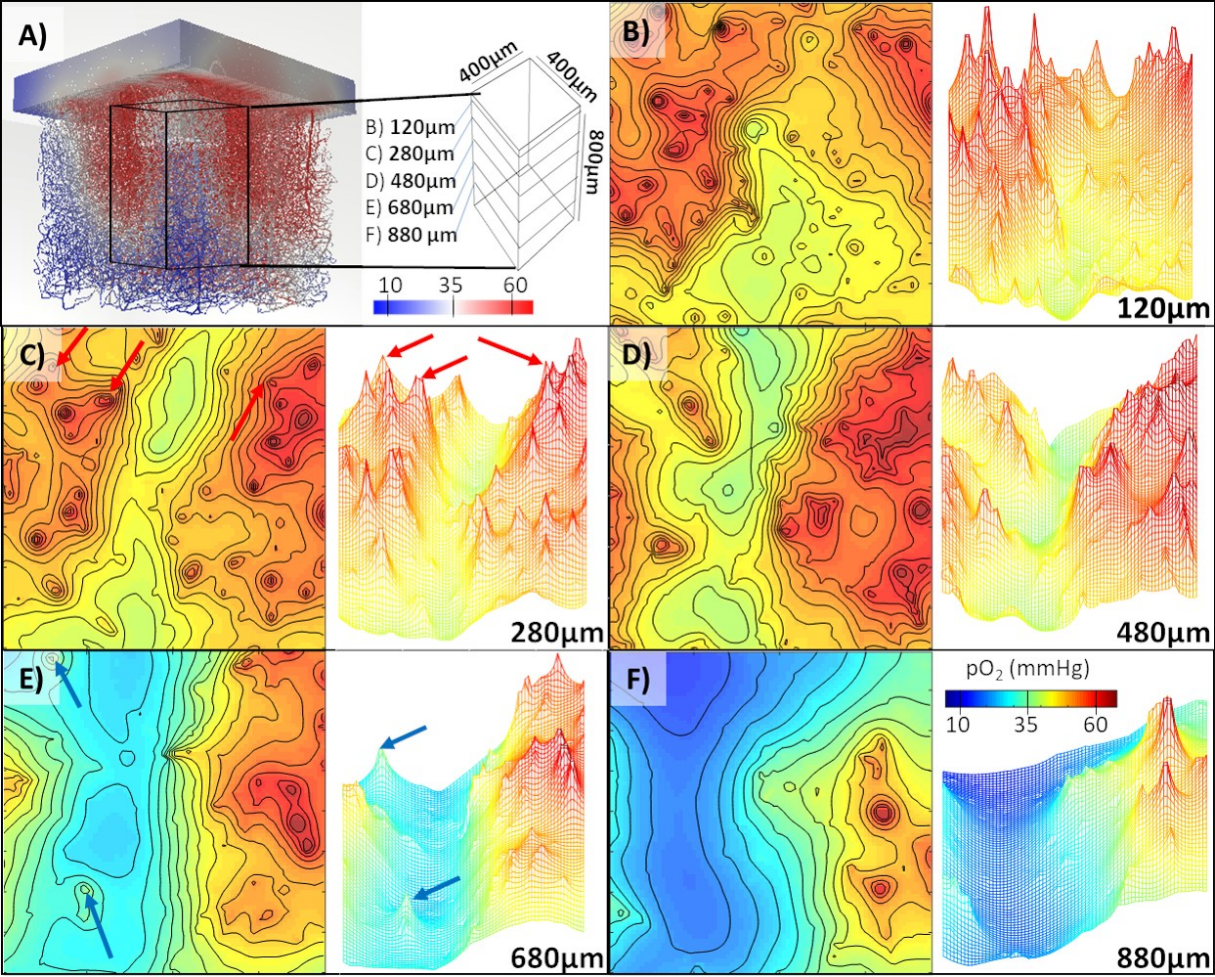
Oxygen distribution throughout a microvascular network specimen (E4.1) from the vibrissa primary sensory cortex in mouse. A) Visualization of a portion of the extravascular space with a block cutout defined by 5 observation planes at different cortical depths (120, 280, 480, 680, and 880μm below the cortical surface). Color coding corresponds to oxygen tension in blood vessels. B-F) The visualization of oxygen distribution at the individual layers. C) Steep oxygen gradients form around penetrating arterioles–marked with red arrows. E) Oxygen tension is also elevated around veins–marked with blue arrows. In the deeper regions E-F) a hypoxic valley begins to form seen as deep blue basins.

We next wished to further analyze the pattern of oxygen dispersion in the extravascular space surrounding the cortical microcirculation. We performed simulations of blood flow and oxygen tension on large sections of the murine somatosensory cortex. To better highlight oxygen profiles around the microcirculation, we created contour maps and pO2 reliefs as shown in Fig 4 for the first specimen. Sharp peaks of high pO2 tension can be seen near oxygen rich penetrating arterioles. Note, that the high-resolution oxygen predictions inside the 3D computational domain also delineate a narrow plateau corresponding to the diameter of the vascular lumen.

Venules are typically located in flat basins of low oxygen tension. Accordingly, areas further away from arterioles but close to venules mark regions with relatively low oxygen tension depicted in blue. Note that the pO2 in venules is typically still above the tissue oxygenation level, which is lowest because of oxygen metabolism takings place inside the cell mitrochondria. We also could confirm that in some locations, venules may reabsorb oxygen from the surrounding tissue. However, our simulations seem to suggest that oxygen drainage is not a typical role for venules.

The results in Fig 4 demonstrate successful simulations for a second cortical dataset and with trends confirming the consistent predictions seen in the first case study. Both case studies also quantified the evolution of the oxygen tension as a function of the cortical depth. For example, in Fig 4D a narrow band of low oxygen tension can be seen at a depth of 880μm below the pial surface. The concentration profile in the vicinity of major vessels exhibit steep oxygen gradients as expected. The precise quantification and detection of low oxygen pockets may be significant for the analysis of pathologies associated with aging.

These simulations show fine-grained resolution of the microgradients surrounding larger vessels while maintaining numerical stability at nPoints>100K vascular nodes, and nVols>8M tissue elements within only two CPU hours on a personal computer (Intel Xeon W-2025, 1 core, 256GB RAM). High resolution of the oxygen field on the micron scale constitutes the desired simulation objective.

### Validation of tissue oxygen predictions against maps from 2 photon imaging

**Fig 5:**
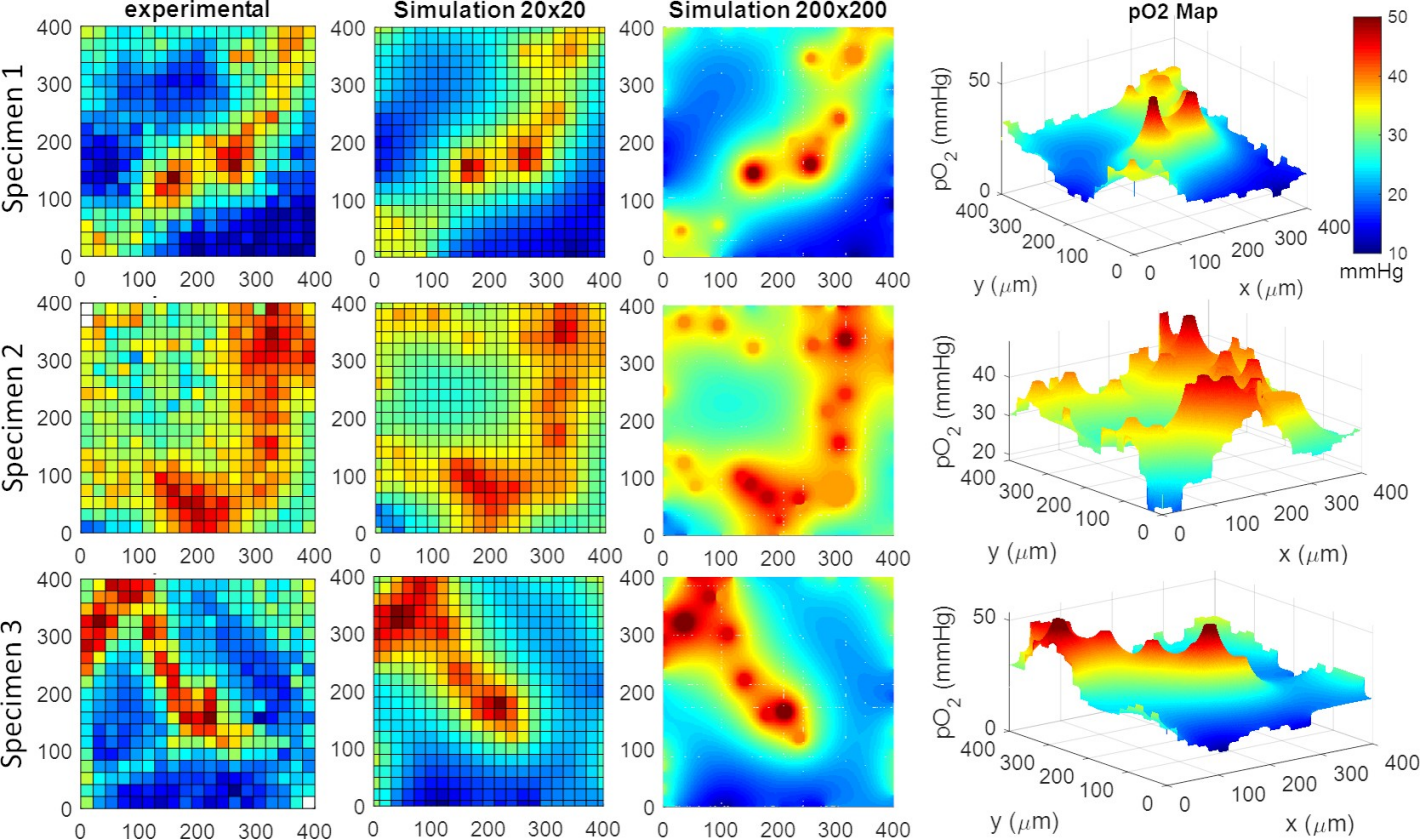
Validation of oxygen perfusion simulations against three sets of experimental data. The experimental results were obtained with 2-photon phosphorescence lifetime oxygen imaging. The simulations were performed in planes with two different resolutions; a coarse 20x20 and a dense 200x200. There is reasonable agreement between experimental and simulated oxygen fields (2.9±1.4% error). Manual matching between the concentration profiles was used to determine tissue diffusivity, reaction rate, and mass transfer coefficient. The 3D perspective illustrates the steep concentration gradients along two penetrating arterioles.

We further tested whether predicted tissue oxygenation fields would match actual in-vivo 2-photon phosphorescence lifetime imaging oxygen measurements. Accordingly, we acquired oxygen tension maps in three mouse specimen at various cortical depths (up to 250μm below the cortical surface) with the acquisition protocol given in the Materials and Methods section. We reconstructed the vascular microarchitecture in these specimen and simulated tissue oxygen tension in several plains below the cortical surface. In all three cases, predictions of oxygen tension utilizing this mesh masking technique shows qualitative and quantitative agreement with the experimental distributions as can be seen in Fig 5. For comparison, we first generated a coarse (20x20 resolution = 400x400μm^2^) visualization using spatial averaging with Cartesian meshes matching the resolution of the coarse image acquisition (experimental data). These side by side comparison shows reasonable agreement between experimental data and simulated oxygen fields (2.9±1.4% error).

We also produced high-resolution simulations (200x200 resolution = 400x400μm^2^) which resolve the oxygen gradient at a much higher resolution than the data in the experiments. The high-resolution simulations also allow us to determine physiological parameters directly from measured oxygen data. Specifically, it is possible to vary diffusivity, D, mass transfer coefficient, U, and even oxygen metabolic rate in the simulation until there is optimal alignment with the experimental oxygen field. This procedure can be interpreted as a multi-dimensional least-square adjustment of unknown physiological parameters directly from experimental data. For the purpose of this demonstration, we performed the adjustment manually with resulting values listed in Table 2. Here, the measured oxygen concentration derived from the original image (= at edges of the field of view and in the center of the blood vessel) served as boundary conditions for the predicted oxygen field. In principle, one could formulate a multi-parametric multi-dimension parameter estimation problem. A mathematical programming solution for the parameter estimation problem is unfortunately outside the scope of this paper.

**Table 2:** Parameters used in the prediction of oxygen tension throughout the murine cortex.

Property	symbol	value	units	Literature	Ref
Diffusivity	D	1,800.00	μm^2^/s	1,800.00	[56]
Transmembrane permeability	U	2,400.00	μm^2^/s	2,400.00	[57]
Metabolic rate	k_1_	14.17	1/ms	3.4–417.0	[14,30]
Endothelial layer thickness	w	1.00	μm	1.00	[36]
Pressure drop (BC)	p	115.00	mmHg	115.00	[27,58]
Concentration inlet (BC)	c	68.50	mmHg	68.50	[58]
Systemic Hematocrit	h	0.35	--	0.35	[27]

### Applications

Two larger scale applications are discussed in this subsection to demonstrate the significance of the proposed technique for assessing age-related metabolic decline and neurodegeneration.

#### Hypoxia in aged brains

The first application concerns a preliminary exploration of age-related changes in the oxygen perfusion of the cerebral cortex. The aged brain suffers from a variety of vascular changes including an increased incidence (~30%) of micro-occlusions [11], systemic hematocrit decrease by ~30% [4], and a ~20% decrease microvascular vessel density [3–8]. It is further believed that these changes may lead to hypoxic pockets, which are regions below pO2<10 mmHg that may suffer damage to neural and glia cell lines. We wanted to test the hypothesis of the effect of aging by simulating oxygen tension in vascular networks, where numerous known age-related morphological or hemodynamic changes were imposed artificially *in silico*. The simulated reduction in oxygen tension was assumed to characterize possible deterioration due to different age-related pathologies.

**Fig 6:**
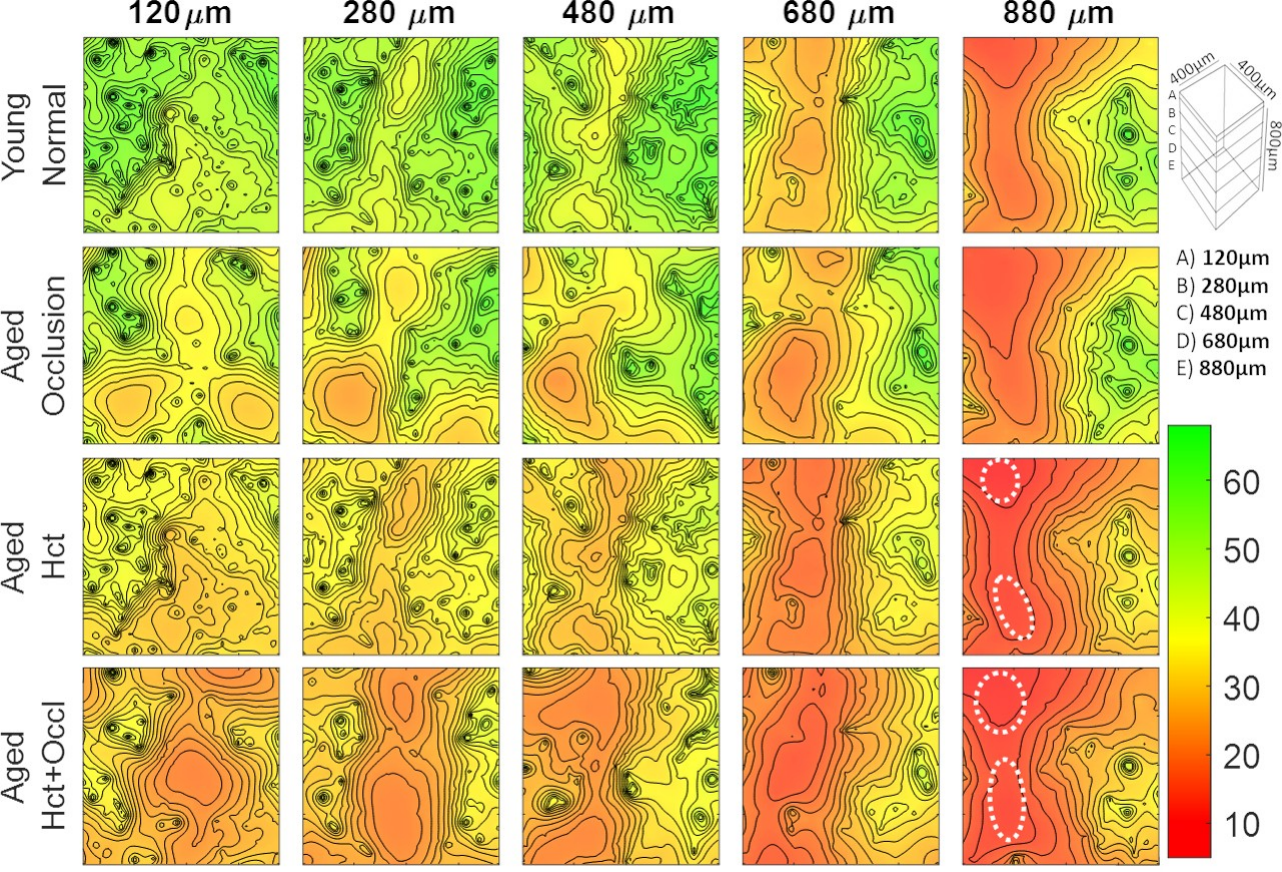
Simulations of age-related changes in oxygen tension at different cortical depths: 120, 280, 480, 680, and 880μm. (Top Row) young normal brain, (Second Row) aged brain with capillary micro-occlusions. (Third Row) aged brain with reduced systemic hematocrit, and (Bottom Row) aged brain with the combined aging effect of reduced hematocrit and micro-occlusions. The region of tissue containing low oxygen content greatly increase in the aged models. Hypoxic pockets form in the lower cortical region when the effect of hematocrit and micro-occlusions combine (aged Hct+Occl at 880μm). The white dotted regions delineate the formation of hypoxic regions.

Quantitative results of the preliminary study on the effects of aging are summarized in Fig 6 which shows comparisons of oxygen perfusion patterns in the normal cortex (young brain, row 1) with different aged brain scenarios (rows 2, 3, and 4). The second row of Fig 6 characterizes the effect of age-related occlusions, which were induced in the simulation by setting the diameter of 30% of the capillaries to a value of numerical zero. Despite the substantial reduction in capillary perfusion, the oxygen tension stayed above the hypoxic threshold for the entire depth of the cortical window (= cortical depth of 880 microns, layer V). In all layers, however, areas of low oxygen are somewhat expanded with the strongest increases in deepest layers.

The effects of reduced systemic hematocrit on oxygen tension in different cortical layers are quantified in row 3 of Fig 6. This simulated aging scenario was realized by reducing the systemic (= inlet) hematocrit by 30% from normal conditions, h_young_ = 0.35 or h_age_ = 0.7x0.35. The observed lower tissue oxygenation in row 3 suggests that systemic hematocrit reduction has a stronger effect on lowering oxygen tension than micro-occlusions.

Finally, the combined effect of reduced hematocrit with increased occurrence of micro-occlusions is quantified in the oxygen maps of row 4 of Fig 6. In this combined effect scenario, hypoxic pockets with oxygen tension under a critical level of pO_2_<10mmHg begin to form especially in the deeper cortical layers (880 microns). The simulations results support the possibility of hypoxic pocket formation due to the combined effects of age-related metabolic decline in the angioarchitecture and hemodynamic functions.

Our simulations demonstrate that high-fidelity computations with the Cartesian meshing are promising for characterizing age-related changes to tissue oxygenation. It should be noted that these results are preliminary and a detailed investigation of age-related changes to cerebral oxygen tension is beyond the scope of this study.

#### Large-scale cortical network simulations to eliminate boundary effects

We observed that the depletion of vascular segments in the boundary zone is a main contributor to boundary effects in oxygen simulations. To overcome this undesired boundary sensitivity, one could artificially seed border regions with artificially added blood vessels so that the vascular density is not disturbed at the domain edge. However, it is not clear which metrics should inform such a “correction” for irregular edge vascularization. A more concise strategy entails generating large samples of the cortical microcirculation so that the domain boundaries are far away from the area of analysis. This idea is demonstrated next.

**Fig 7:**
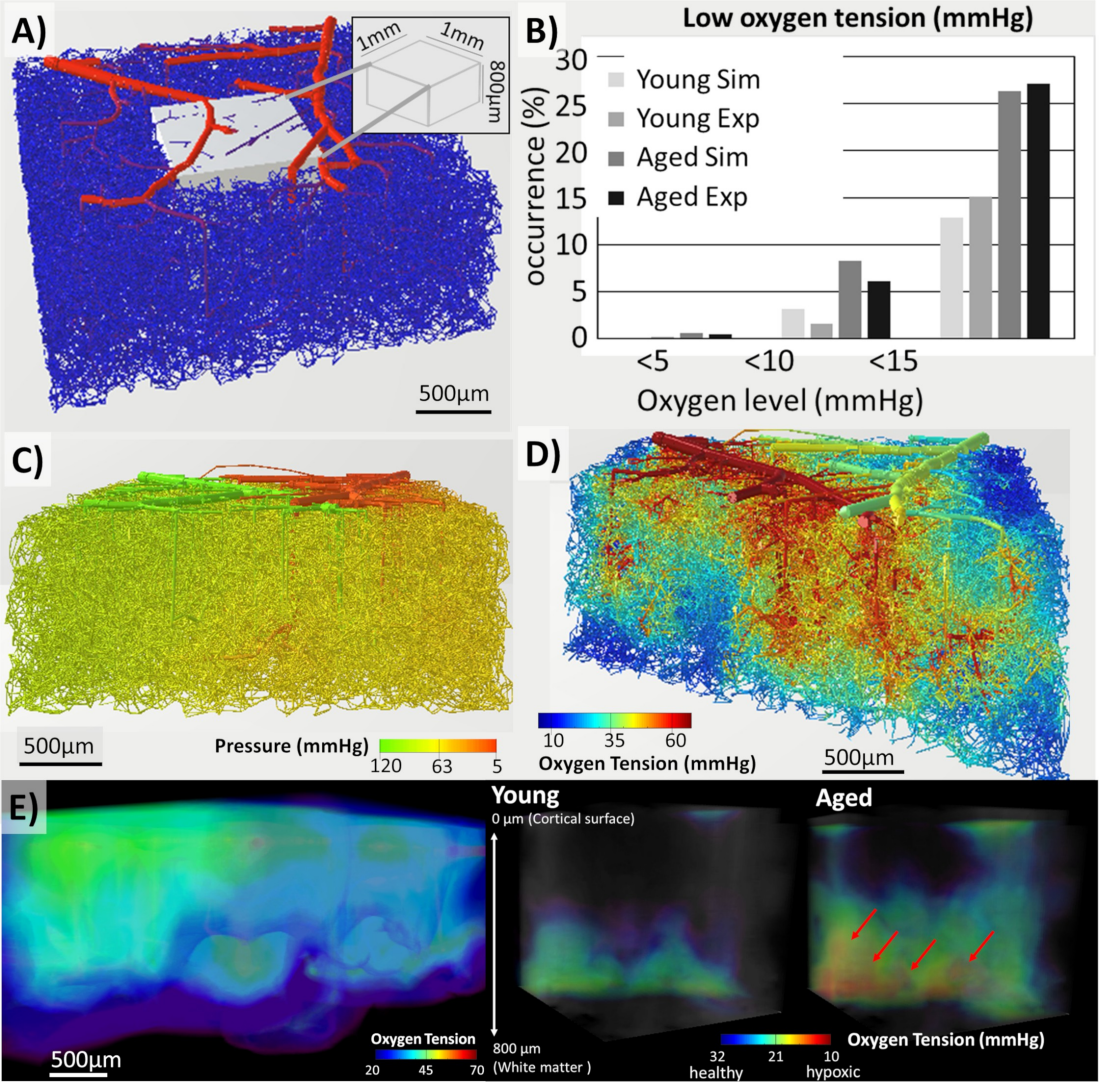
Comparison between oxygen tension in young mouse and aged mouse suffering from reduced hematocrit in a large size synthetic vascular network spanning 3x3x1.2mm^3^ of the mouse cortex. A) The vascular structure colored by diameter (red = thick, blue = thin) shows a dense set of tortuous vessels connected through a complete capillary bed. B) The tabulated occurrence rate of low-oxygen mesh elements in a portion (= a 1x1x0.8mm^3^ tissue subset from the interior of the domain) of the predicted oxygen field shows the same trends as in the experimental data. C) Predicted pressure distribution throughout the network matches the topology of arteries, veins, and capillary closure. D) A cut through the dense structure reveals regions of hypoxia and pockets of hypoxia occur more frequently in the lower regions of the cortex. D) A zoomed view of oxygen tension in the interior of the vascular network. There are some segments with low oxygen content (but above the hypoxic threshold) especially in deeper regions). E) The oxygen levels in the tissue reveal the lower regions are more susceptible to lower oxygen tension then regions near the cortical surface. A detailed view compares oxygen tension in young to aged specimen. In the young specimen, no hypoxic areas form. In the simulated age mouse, hypoxic regions and their spatial extent were identified; hypoxic pockets are indicated in red and are larger and more prevalent in the aged brain.

We created a large artificial vascular network by using an *image-based circulatory network synthesis* (iCNS) algorithm which is described in detail elsewhere [52,59]. Specifically, we synthesized a topologically equivalent murine microcirculatory network sample covering 9mm^2^ of its cortical surface to a depth of 1.2mm. The resulting microcirculatory network model depicted in Fig 7 is roughly one order of magnitude larger than the largest 2-photon Kleinfeld data set, and much larger than samples of previous studies. We limited our analysis to the inner region of interest (ROI) which fits into a box of 1x1x0.8mm^3^ and is perfused by an uninterrupted, physiologically-consistent microcirculatory network with domain borders so far removed that they do not affect the oxygen simulation in the ROI. We conducted blood flow and oxygen simulations over this massive network with *nPts*~1M network segments and *nVols*~100M mesh elements. The Cartesian masking method converges stably for this massive domain sizes in ~28 CPU hours on a personal PC (Intel Xeon W-2125 processor at 4.01GHz and 256 GB of Micron MTA36ASF4G72PZ-2G3B1 memory). Fig 7E shows the mesh domain, but also the unavoidable boundary effects at corner, bottom, and side edges of the domain. The calculated oxygen fields in the ROI showed no observable boundary effects.

For this massive cortical sample, we also simulated the effect of aging by the combined effect of reducing the inlet hematocrit and imposing micro-occlusions in 30% of the capillaries as described above. Inside the ROI, we found that young normal brains exhibited no areas with critically low oxygen tension (= young brain, Fig 7C). In contrast, multiple hypoxic pockets with oxygen concentration below the critical pO2<10mmHg threshold formed in the aged brain. An example of the spatial extent of a hypoxic pocket is depicted in Fig 7D.

We also computed the volume fraction occupied by mild hypoxic regions (pO2<10mmHg) and tabulated the increase in occurrence for cohorts of normal (N = 7) to aged (N = 7) murine brains in Fig 7B. The simulated values were compared to detailed experimental results obtained in a different study using two-photon phosphorescence lifetime imaging [13], which studies oxygen tension in cohorts of young, middle aged and old mice. The preliminary comparative data show that the simulations matches the incidence of low oxygen regions in the normal brain. Moreover, the incidence of severe hypoxic pockets (= regions with oxygen tension less than 5mmHg) observed in the different age cohorts is closely matched in the massive network simulations presented here. The occurrence of regions in aged brains where oxygen tension was less than 10 and 15mmHg respectively were drastically increased in the aged population, both in the experiment as well as in our large-scale computer simulations.

## Discussion

*Voxelized simulation domain*. The proposed “voxelized” Cartesian meshing-free methodology offers a robust approach for stable convergence of blood flow and oxygen exchange simulations for very large, anatomically detailed sections of the cerebral cortex. This method succeeded in predicting blood and oxygen micro-perfusion in massive cortical microcirculatory networks with stable convergence up to relatively dense mesh resolutions in the micron range.

The use of a voxelized grid with cubic elements (= cuboids) delivered two critical benefits: First, a mask based logic introduces a structured Cartesian grid representation which resolves the interface between the endothelium and the extravascular space without the need of manual body-fitted segmentation required for tetrahedral grids. Therefore, we termed the method *mesh generation-free*, *which means that it does not require the creation of a body fitted mesh*. Second, the *stencil* of the resulting Cartesian mesh always contains exactly seven cells, so that the bandwidth of the coupled mass transfer problem is always much smaller than in tetrahedral grids. Moreover, since the Cartesian meshes are structured and parametric in the main coordinate directions (x, y, z-directions) leading to a mesh comprised entirely of uniform cuboid elements, there is no need to store the tissue domain grid, in effect drastically lowering the memory requirements of the simulation code. That property also justifies the naming of a technique that is mesh-free, because it does not require the cumbersome generation of memory expensive fitted meshes. The compact structured cubic mesh with narrower matrix bandwidth reduces problem size, stabilizes the perfusion problem against inaccurate initial guesses and drastically accelerates convergence. The drastic improvements in the structural matrix properties of the transport problem were illustrated in Fig 1. The proposed binary masking technique also enables automatic assignment of mechanistic expressions for oxygen transfer fluxes between vascular segments and the adjacent tissue elements through a *fuzzy* endothelial cell layer. The sharply defined interface obtained with this technique does not introduce singularities in the mass transfer flux computations. In other numerical techniques, singularities may occur when a massive flux discharges into a small volume cell that cannot accommodate the flux without incurring an extreme concentration drop. This does not occur in the current discretization technique, because large mass exchange fluxes emanating from thicker vessels are partitioned and distributed across the endothelial interface in proportion to the tissue cell edge length. In other words, the massive oxygen efflux is divided among the set of adjacent cuboids in agreement with the characteristic edge length thus avoiding singular concentration gradients.

Moreover, proposed “voxelized” simulation domain facilitates the direct comparison to stacked slices of neuroimage data. Thus, microcirculation simulations can be conveniently set up directly from image stacks without the need of first segmenting the vasculature-tissue interface with a body-fitted tetrahedral grids, a task that is currently infeasible for automatic mesh generation software.

*Boundary effects*. Lorthois [15,60] pointed out that boundary conditions severely affect microcirculatory hemodynamic predictions. In their microcircular blood flow simulations, Schmid et. al [61] assembled an array of cloned samples to limit border effects. Gould et al [27] cyclically extended the tissue space with vascular segment connections across opposing domain borders to diminish boundary effects. In the current study, we observed that boundary effects in oxygen tension computations mainly stem from the fact that neuroimaging data typically do not capture vascular topology accurately at the edge of the imaging domain. This irregularity especially affects branches of large penetrating arteriole trees (causing dangling segments that often have to be removed). The removal causes the boundary region in simulations to appear undersupplied with oxygen carrying blood vessels, thus creating a marked disturbance of oxygen perfusion in the border zone. To overcome this undesirable disruption, one could populate the boundary region with artificial blood vessels so as to compensate for the severed segments at the domain edge. However, it is not clear which metrics should inform the “correction” for irregular edge vascularization. Our more concise solution strategy entails generating large samples of the cortical microcirculation so that the domain borders are distant from the area of interest.

We applied an *image-based cerebrovascular network synthesis* (iCNS) algorithm [59] to create a replica of a sizable portion of the murine somatosensory cortex with dimensions of 3x3x1.2mm^3^. Note that these dimensions are much larger than previously reported microcirculatory oxygen simulation domains. Then we limited our analysis to a region of interest (ROI) at the interior core. The ROI measuring only 1x1x0.8mm^3^ has all its sides far removed from domain boundaries. Edge effects in oxygen supply due to the disruption of the integrity of the vascular bed near the boundary were thus kept away from the predicted oxygen fields. However, expanding the computational domain to eliminate boundary effects necessarily requires the large-scale solution of oxygen perfusion problems which is fortunately enabled by the proposed voxelized simulation technique.

*Multiscale acceleration*. Linear algebraic system equations for ultra-dense grids (= massive cell numbers) do not always converge from poor initial guesses. Moreover, the numbers of iterations to achieve convergence may be prohibitive due to the deterioration of the system condition number that comes about with fine mesh resolutions as was mentioned in the introduction and is discussed in detail elsewhere [39].

To ensure robust convergence for arbitrary initial values, we took advantage of multiscaling effects by obtaining approximate oxygen fields initially on a coarse mesh, then computing intermediate updates on Cartesian grids with finer resolution, before converging the final solutions at the finest scale. The masking procedure needed for meshes of different resolution can be performed easily by simply setting the edge length to a smaller value. Coarse-grain simulations rapidly converged due to their reduced problem size, but lacked details such as local oxygen micro-gradients. Computed coarse-grain oxygen fields were then linearly interpolated down to the finer partitions (= finite element interpolation) and used as starting conditions for fine grid simulations. In all cases, we succeeded in converging from uninformed, of “poor”, initial guesses where frontal fine-grained attempts tend to diverge.

Our method differs from classical *deflation* as it does not solve dual-stage problems using the residual vectors to compute reduced-order updates, but merely uses consecutively more refine-grid solutions as better initial guesses. In its current form, our method does not benefit from such accelerated convergence methods, but does seem to have a stabilizing effect by suppressing high frequency updates and preventing them from overtaking the solution.

An interesting, unexplored approach using our *mesh generation-free* Cartesian technique would be to combine it with deflation as proposed by Nicolaides [62]. It can be expected that the geometric (= positions in 3D space) and the logical regularity of the Cartesian mesh (= block diagonal arrangements of matrix entries) tremendously aides in the implementation of geometric multigrid techniques [39,63]. The numerical accuracy of proposed computational technique might be further improved with the use of logarithmic profile assumptions [35]. We did not yet attempt to deploy geometric multigrid algorithms [64] with the structured meshing-less perfusion problem formulations described in this manuscript, although these can be used in the future. Reported convergence could be further accelerated by algebraic multigrid solver algorithms [65] to further performance gains, but these ideas are outside the scope of this study.

*Fuzzy 3D blood flow computation*. We also tried to solve blood flow and oxygen convection over the “voxelized” 3D vascular grid at differing resolutions but found this to be unstable in our implementation [66]. Thus, we avoided numerical problems with frontal 3D blood flow computations by instead, merely solving the blood flow and pressure fields once over the original network (before segmentation). The fluxes and pressure fields were interpolated to assign the hemodynamic states for segment subdivisions.

*Comparison to prior methods*. To better assess the significance of the proposed technique, a selection of representative prior work of microcirculatory simulations are compared in Table 3. The list is not complete and many valuable contributions could not be incorporated due to space limitations; specifically we did not list *homogenization* techniques [46,47] because they do not currently address oxygen simulations, but approximate capillary blood perfusion using a continuum surrogate for the microvessels. The proposed “voxelized” method compared favorably in problem size as measured by the number of vascular segments and mesh elements in relation to previous techniques. The simulation time for the E4.1 data set (with a resolution of 305x305x305 elements) was ~1 CPU hour and the simulation time for the 3x3x1 (with 455x455x455 mesh elements) was 19.4 CPU hours. Note, the current simulations of the microcirculatory blood flow and oxygen fields used an unprecedented fine grid resolution (= micron scale).

**Table 3:** Comparison of simulation methods for blood and oxygen microperfusion.

Model	Tissue domain size (mm^3^)	Dimensions (μm)	Number of vascular segments (nPts)	Number of mesh elements (nVol)	CPU time (hrs)	Solver type	Ref
Dual mesh	0.011	360x200x150	12	*NA*	*NA*	*NA*	[33]
Domain splitting	0.011	350x200x150	~30	*NA*	*NA*	*NA*	[33]
Singularity removal	*NA*	*NA*	~3,000	*NA*	*NA*	Iterative	[35]
Green’s function	0.066	550x520x230	~100	2,754	>0.28**	Iterative*	[31]
Dual mesh	0.125	500x500x500	250	~400,000	0.5	Iterative	[42]
Dual mesh	27.000	3000x3000x3000	256,000	600,000	<0.13	Direct	[36]
Body fitted tetra	0.024	230x230x450	~2000	607,406	0.04**	*NA*	[30]
Dual mesh	2.709	1345x1456x1383	33,108	~3,000,000	~24.00	Iterative	[27]
**This work (1x1x1)**	2.709	1345x1456x1383	33,108	28,930,775	2.58	Iterative	-
FEniCS tetra	0.06e-6	~4x4x4	0	84,000,000	>1000.00	Iterative	[37]
**This work (3x3x1)**	10.800	3000x3000x1200	964,430	95,438,525	19.40	Iterative	-

NA indicates the values were not reported

** Time represents a single iteration. Many steps are required (many multiples of this value)

* Only solves for the blood oxygen, not tissue

Analytic and semi-analytic approaches that were introduced in the Introduction section resolve the oxygen exchange fluxes, nested infinite series approximating the oxygen fields for the vascular network and the extravascular mesh domain have to be repeatedly evaluated until convergence is achieved. Accordingly, the computational complexity grows considerably with the number of source points (= vascular segments). So far, oxygen exchange for networks with a few hundred segments have been demonstrated [31,32,35]. The reported problem sizes should be seen in relation to the required vascular density of ~11,600 vascular segments/mm^3^ of tissue. The second semi-analytical method in this group is splitting. Gjerde et al [35] substantially reduced the computational effort by separating the solution into an analytic base component and a *corrective* field, which needs to be determined numerically. Although this offers considerable performance gains, these methods are mathematically intractable for networks at the scale we use here.

Other groups used a dual-mesh technique for modeling water exchange on bifurcating vascular models up to a few dozen segments with variable permeability to simulate the difference between healthy and diseased capillaries [67]. To avoid singularities from the 1D-3D coupling, the authors distributed extravascular discharge flux using a nonuniform shape function, which was able to stabilize solvability even at coarse grid resolutions [68]. Shape functions are also used in algorithms to discharge the vascular flux into the homogenized domain [46]. Other groups developed sophisticated methods that coregistered the circumference of the tangential disc representing each cylinder with the surrounding tetrahedral mesh [33,34,40–43] to avoid singularity issues. High quality dense meshing is required to effectively resolve gradients surrounding the smallest capillaries, so that adaptive meshing becomes necessary [33]. Despite their mathematical elegance, simulations for sizeable sections of the cortical microcirculation have so far eluded these techniques due to their fine micro-resolution requirement.

It is critical to underscore the benefits of the computational approach with simulations on a domain sizes comparable to in vivo image data. The results here demonstrate several key innovations:

- The ability to solve flow and oxygen exchange over substantial domain sizes enables validation of predicted oxygen gradients and fields against in vivo multiphoton image data. While several prior approaches laid out elegant mathematical foundations for highly accurate 1D-3D coupling computations, these approaches engender intractable problem sizes when bridging the micro to macroscales characteristic of perfusion in the cerebral circulation.
- Successful convergence of massive problems over domains that are large enough that their boundaries are far removed from the region of interest. Our simulations further show that oxygen perfusion simulations over domains smaller than 1x1x1mm^3^ are severely affected by boundary choices, so that predicted results may be excessively affect the simulated metabolic conditions instead of physiological oxygen exchange. Severe boundary effects are depicted in the oxygen field simulations shown in S4 Text.
- The simulations were highly sensitive allowing the prediction of subtle perfusion changes related to age-related decline. Specifically, we are able to confirm the experimental discovery of hypoxic pockets acquired with in vivo multiphoton observations, and predict with fidelity size and location of hypoxic tissue locations.

*Limitations*. The preliminary results reported in this manuscript on the effects of aging should not be interpreted as in-depth physiologically validated conclusions about the aging brain. The study merely aimed at showing that the proposed method is applicable to problem size with fine-grid resolution needed to quantify even subtle effects of age-related hemodynamic changes on tissue oxygen tension. A separate study to investigate the mechanistic sources of age-related oxygen tension changes is in preparation.

Furthermore, we would like to point out that the change in oxygen extraction also impacts the tissue pH which further feeds back on the kinetics of the oxygen dissociation from hemoglobin to blood plasma [69,70]. Without accounting for pH effects, the predicted oxygen tension may be inconsistent with the complete physiological response seen in vivo. However, we are currently not aware of any methodology that would simultaneously solve for blood flow, hematocrit, oxygenation, dissociation kinetics, oxygen and CO_2_ metabolism and its effect on the pH Accordingly, we defer the inclusion of pH changes to future work.

We also note that the assumed pressure drop across the microcirculatory network of Δp = 115mmHg is somewhat large. We have pointed out previously [27,52,58] that our simulations of the Kleinfeld dataset use the original diameter specification which may report some diameters in the smallest capillaries too narrow so that larger pressure driving forces are needed to obtain physiological flow rates.

*Conclusions*. In conclusion, the proposed *voxelized* technique which requires no *mesh generation* offers a scalable method for performing computer simulations of solute exchange and metabolism for sizable sections of the cerebral cortex. The numerical techniques presented here compliment recent advancements in microcirculatory network synthesis and simulation [52,59]. A combined approach that integrates neuroimage data from multiple sources into mechanistic perfusion simulations of the mouse cortex was illustrated. Several anatomically detailed applications showed stable mesh convergence up to ~100 million equations. It should be easy to further expand this limit, which in our case studies was limited only due to the memory capacity of our local personal computers. Predictions derived from anatomically detailed mechanistic models are expected to be useful for testing medical hypotheses for normal or pathological states.

## Materials and methods

### Ethics statement

All protocols executed at the University of California, San Diego were approved by the Institutional Animal Care and Use Committee at University of California, San Diego. Animal handling and surgical procedures performed at the Montreal Heart Institute were approved by the ethics committee of the research center of the Montreal Heart Institute. All experiments at the Montreal Heart Institute were performed in accordance with the Canadian Council on Animal Care recommendations.

### Vascular structure acquisition

Four sections of the murine vibrissa primary sensory cortex [53] were reconstructed from two-photon laser scanning microscopy (2PLSM) images. This dataset is referred to in this paper as the *Kleinfeld* data set. These images represented the length and orientation of the blood vessels [53,71,72]. These structures were then labeled with an automated algorithm as described in the original manuscript. The categorization used size and branching level information (Strahler order) to differentiate between pial vessels, penetrators and capillaries. Capillaries were identified by a diameter cutoff of 6 μm and penetrating vessels differentiated from pial vessels with depth and diameter thresholds. The final reconstructed network topology and diameter information was stored using sparse connectivity matrices. More details on image acquisition [53,71,72], image reconstruction [73], as well as the formulation of the network equations [58] can be found elsewhere. All protocols were approved by the Institutional Animal Care and Use Committee at University of California, San Diego.

### Oxygen tension measurements in young and aged brain

Data acquisition and methods are described in the original experimental study reported in [13]. In short, the O2-sensitive molecule PtP-C343 [74] was used to measure tissue oxygen in awake animals using a cranial window and head fixation. Following fixation, two-photon imaging was performed using a custom-built laser-scanning microscope that used a consecutive sequence of 820 nm, 80 MHz, 150 fs pulses through an electro-optic modulator (ConOptics, USA) to adjust the gain and allow the generation of alternating *on* and *off* laser pulse periods for microsecond lifetime imaging required for oxygen quantification. Cortical tissue oxygen data was acquired in young, middle aged, and old mice, scanning 400um x 400um adjacent planes to estimate O2 in large areas. Twenty (20) mice were used for tissue oxygen measurements, 7 young (8.8±0.1 months), 6 middle-aged (15.3±0.1 months) and 7 old (27±0.1 months) at various depths up to 250–300μm below the cortical surface. Animal handling and surgical procedures were approved by the ethics committee of the research center of the Montreal Heart Institute. All experiments were performed in accordance with the Canadian Council on Animal Care recommendations.

### Mathematical blood flow and oxygen model

Blood flow and hematocrit were computed first, then the oxygen fields in the vasculature and the tissue are solved with the blood and hematocrit fields as inputs.

*Blood flow*. Blood flow is modeled as a biphasic suspension with details in [26,52] and summarized in system (1)–(2), which has nonlinear coupling between the flow, *f*, pressure, *p*, and hematocrit fields, *h*. To solve the coupled flow problems efficiently, we perform fixed point iteration which computes the flow rate, *f*, using the diameter dependent resistance matrix, *R*, for an assumed hematocrit field, *h*. Approximate blood flows can then be computed with (1) using linear algebraic methods. With the converged flow field, *f*, the linear subsystem for hematocrit in (2) is solved consecutively to update the prior hematocrit field. Hematocrit updates are in-turn used to recalculate the flow field in (1) until the two fields converge. In our implementation convergence is typically achieved in less than 30 iterations.

$$\left[\begin{array}{cc} R(h) & {-C}_{1}\\ \left(I-D)[C_{1}^{T}(f,h)\right] & D \end{array}\right]\left(\begin{array}{c} f\\ p \end{array}\right)=\left(\begin{array}{c} 0\\ D\bar{p} \end{array}\right)+\left(\begin{array}{c} 0\\ (I-D)\bar{q} \end{array}\right)$$ (1)

$$\left[{\bar{M}}_{c}(f,h)][h]=[D_{0}\bar{h}\right]$$ (2)

Here, $C_{1}\in R^{nArcs\;x\;nPts}$ is the fundamental incident matrix (= arc connectivity matrix) as expressed by Tellegen’s theorem [75] and $R\in R^{nArcs\;x\;nArcs}$ is the matrix of vessel segment resistances. Eq (2) is used to compute the hematocrit field with plasma skimming [52,58].

More details can be found in [26,52,59]. The matrix ${\bar{M}}_{c}\in R^{nPts\;x\;nPts}$ encodes hematocrit convection with $\bar{h}$ given inlet boundary conditions enforced by the decision matrix $D_{0}\in R^{nVol\;x\;nVol}$. The counter *nArcs* indicates the number of vascular segments, and *nPts* is the number of vascular nodes.

*Oxygen field*. The blood flow, *f*, and hematocrit fields, *h*, computed from (1)–(2) serve as inputs to a convection-advection oxygen transport model with metabolic reactions in brain tissue. The final system is summarized in simplified conceptual matrix form in (3), which we solve simultaneously for oxygen tension in blood vessels, $c_{v}\in R^{nPts}$, and in tissue, $c_{t}{\in R}^{nVol}$. Oxygenated blood enters the cortical microcircuitry through pial arteries; oxygen travels by convection through the microcirculatory network and exits through the pial venous outlets, $M_{c}\in R^{nPts\;x\;nPts}$. Oxygen also transfers across the blood brain barrier (BBB) into the tissue (= extravascular space), where it metabolizes to satisfy the energy demand of neurons and glial cells. The connectivity matrix $C_{3}\in R^{nPts\;x\;nPts+nVol}$ enforces the mass transfer between the vascular nodes and endothelial mesh elements which is scaled by the mass transfer conductivity, $\Gamma_{1}=\frac{U\;A}{w}\in R^{nPts+nVol\;x\;nPts+nVol}$. The matrix $M_{d}\in R^{nVol\;x\;nVol}$ encodes the diffusion in endothelial and extravascular (tissue) mesh elements. The diagonal matrix *R*_2_ is used to implement the cerebral oxygen demand (CMRO_2_) and contains the reaction rate constants, *k*_*met*_, and the cuboid volume (*V*_*t*_).

$$\left([\begin{array}{cc} M_{c}(f,h) & 0\\ 0 & 0 \end{array}]-[C_{3}^{T}\Gamma_{1}C_{3}]+[\begin{array}{cc} 0 & 0\\ 0 & M_{d} \end{array}]-[\begin{array}{cc} 0 & 0\\ 0 & R_{2} \end{array}]\right)\left(\begin{array}{c} c_{v}\\ c_{t} \end{array}\right)=\left[\begin{array}{c} D_{1}\bar{c_{v}}\\ D_{2}\bar{c_{t}} \end{array}\right]$$ (3)

In system (3), $\bar{c_{t}}$ and $\bar{c_{v}}$ are the known Dirichlet boundary conditions in the tissue and vasculature respectively aided by the decision matrices $D_{1}\in R^{nPts\;x\;nPts}$ and $D_{2}\in R^{nVol\;x\;nVol}$. In the simplified notation of (3), we show the reduced system in which the interior vessel lumen cells have been eliminated (by identity with the vascular graph). More implementation details are offered in S1 Text. The coupled oxygen problem is further presented in component form for better description of the system equations as follows:

*Oxygen transport in the microvasculature*. According to mass conservation, the change in convective oxygen flux (= the divergence of oxygen convection) carried by the blood stream must be equal to oxygen transfer to the tissue as in (4). As shown above, this relation can be conveniently expressed with the connectivity matrices with the added mass transfer from the vasculature into the tissue. Here, *nVol*, stands for the number of Cartesian tissue volumes (= cuboids). Moreover, *U* is the transmembrane permeability of the endothelial layer, *w* is the endothelial layer thickness, *A* is the endothelial layer surface area.

$$-\overset{⃑}{\nabla }∙[f(h)c_{v}]-U\;A\frac{c_{v}-c_{t}}{w}=0$$ (4)

*Oxygen transport in tissue*. Oxygen transported to the tissue domain is permitted to further disperse by diffusion or undergo cellular reactions according to the cerebral oxygen metabolism in (5). The tissue oxygenation model is a *diffusion-reaction* system with coupled mass transfer sources from the vasculature. The parameters here are; *D*, the isotropic molecular diffusivity and, *k*, the first order cerebral metabolic rate of oxygen (CMRO_2_). In extravascular mesh elements (labeled as blue in Fig 1) there is diffusion and reactions. In the endothelial cells (grey in Fig 1), there is in addition mass exchange with the vascular network. Note, we chose to neglect intravascular diffusion (= diffusion inside the blood vessel lumen), because the magnitude of diffusion is significantly smaller than advective fluxes (Peclet number >> 1) in the axial direction. Inclusion of a radial diffusion term would be possible, but was not undertaken here, because the required continuum assumption of radial diffusion in single phase does not match physiological discrete RBC interaction with the glycoclyx, so we felt justified to neglect radial diffusion at the microscale. Thus, radial concentration profiles were not resolved in our model.

$$\overset{⃑}{\nabla }∙(D\overset{⃑}{\nabla }c_{t})+U\;A\frac{c_{v}-c_{t}}{w}-k\;c_{t}\;V_{t}=0$$ (5)

Note that system (3) above is a simplified concatenated version of the component form of the vascular in (4) and the tissue equations in (5). We also note that the intravascular mesh elements (labeled as red hexahedrons in Fig 1) do not participate in mass transfer, diffusion, or reactions, but can be eliminated from the system by the equality with the associated vascular network node as in (6).

$$c_{t}^{i}=c_{v}^{j}$$ (6)

Where $c_{t}^{i}$ is the concentration of the tissue element and $c_{v}^{j}$ is the concentration of the nearest vascular node.

All physiological parameters and boundary conditions are similar to previously reported values [27,36,58] for oxygen in the brain as listed by Table 2. These values were estimated from experimental data and were found within the range of reported values.

### Automatic equation generation and solution

**Fig 8:**
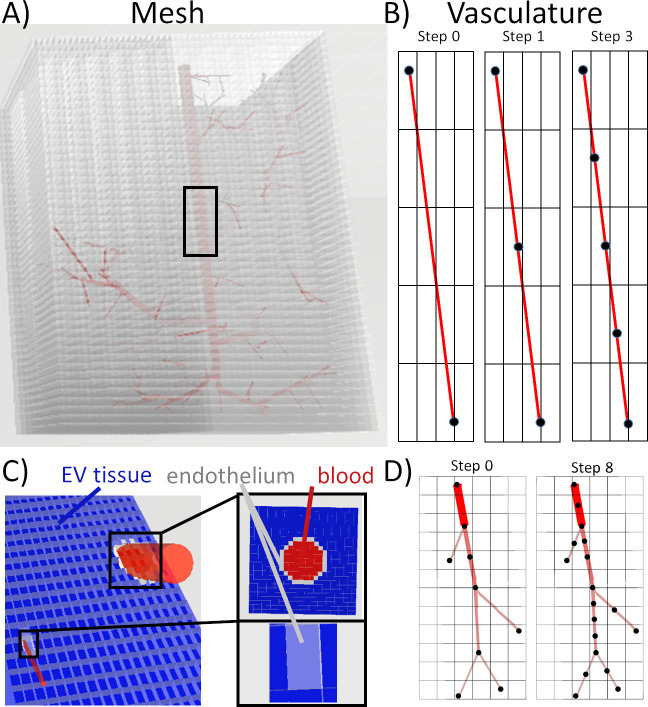
Vascular masking of the voxelized Cartesian mesh and vessel partitioning. A) A penetrating arterial tree with the overlay of a Cartesian mesh reflects a network whose straight segments span many cuboid cell volumes. B) The vasculature is re-segmented (partitioned) until all segments span no more than two adjacent volumes. C) A labeled vessel inside a Cartesian mesh shows the extravascular elements in blue, intravascular segments in red, and endothelial segments in grey. D) The original centerline before and after partitioning for a penetrating arteriole sub-tree. In this illustrative case, the algorithm requires 8 steps to fully partition the tree.

A masking procedure with edge detection logic was applied to identify connectivity between the two domains. We first produce similar characteristic lengths between the Cartesian cuboids and vascular network segments by re-segmenting the vasculature as described in S1 Text. By re-segmenting the vascular network, we ensure the vascular segments have a similar characteristic length as the edges of the cuboid mesh. This stabilizes the simulation, ensuring the vascular segments are able to display realistic axial gradients and that the mass transfer between the vasculature and the surrounding tissue is sensitive to these gradients. An example of partitioning vasculature into many sub-segments is offered in Fig 8B–8D. The process then undergoes two important steps: (i) masking and (ii) equation generation.

Step-1. Masking. The simple fuzzy algorithm *label3DMesh* traverses every vascular segment to identify a bounding box for the centerline as described in S1 Text. To ensure full enclosure of the entire cylindrical segment, the bounding box is then expanded by the width of the segment radius. Once the set of boundary indices is determined, the indices of this subdomain of cells are temporarily stored, denoted as *vesselNeighborhood*. For each cell in the *vesselNeighborhood*, the Euclidean distance, *d*, to the vessel centerline is calculated. A cell is labeled as an *edge cell*, if its center lies inside the endothelial layer defined by a thickness, *w*/2, from the vessel edge. It is labeled as an *interior cell*, if it is not an edge volume and *d*<*r*. Otherwise, a cell is labeled an *extravascular cell*, which is also the default assignment. The pseudocode for masking the *vesselNeighborhood* is listed in S1 Text. An example of mesh labeling is offered in Fig 8A–8C.

Step-2. Equation generation. Once all cells of the Cartesian domain are labeled and connectivity matrices defined as described in S1 Text, we begin with generating the mass balance equations for each element in the simulation domain. First, we assembled the convection matrix for the vascular network (*M*_*c*_) using an upwinding scheme. For its assembly, it is beneficial to reuse incidence matrix of the vascular network (*C*_1_). Furthermore, *M*_*c*_, is asymmetric because of upwinding of the flow field. We did not account for oxygen convection by interstitial water fluxes in the extravascular domain, because such convective transport is not known to play a role in tissue oxygenation.

We then assembled the mass transfer connectivity matrix, (*C*_3_) which connects the graph network nodes to the endothelial mesh cells. The connectivity information is obtained by the masking procedure as described in S1 Text and shown in Fig 2. When multiplying *C*_3_ with the diagonal matrix *G*_1_, we obtain the mass transfer flux balances. The mass transfer flux balance is then accordingly $C_{3}^{T}{G_{1}C}_{3}$, forming a symmetric submatrix system. This mass transfer submatrix also shows structurally very nicely the coupling between the network graph and the Cartesian tissue mesh in our dual-mesh technique.

The diffusion in the tissue is enforced using the diffusion matrix *M*_*d*_. More details on how to assemble the diffusion matrix can be found in S1 Text. Note, the diffusion submatrix for diffusion is symmetric.

The oxygen consumption in the tissue is implemented with the help of the diagonal reactivity matrix *R*_2_. Oxygen consumption in the fourth submatrix is also symmetric.

Finally, on the right hand side, we show conceptually the implementation of boundary conditions for the vascular nodes, $\bar{c_{v}}$, and for the tissue domain, $\bar{c_{t}}$. Here the sparse decision matrices *D*_1_ and *D*_2_ formally indicate those nodes where known boundary values (Dirichlet boundary condition) need to be enforced. Neumann flux boundary conditions can easily be implemented with the logic indicated in (1), but has been omitted here to avoid detraction from the main structure of the overall coupled mass transfer system.

#### Implementation note

In the formulation of (3) we have chosen not to distinguish the tissue from the endothelial nodes so as to avoid clutter in the equation. However, the connectivity matrix of the mass transfer correctly associates network nodes *c*_*v*_ with endothelial nodes. Moreover, we have also omitted the equality between interior vascular nodes on the Cartesian grid and the associated network graph nodes (*c*_*v*_) as in (6). We did not include the interior vascular lumen nodes because they can be eliminated from the system altogether, at which point (3) describes the reduced system. More details given in S1 Text.

All mesh cells in the tissue domain undergo metabolic reactions, *R*_2_ matrix. Very small segments that are entirely embedded in a single extravascular cell exchange oxygen with this one cell only. This happened in the smallest segments of the capillary bed, depending on the Cartesian mesh edge length. In general, numerous cells delineate the endothelial layer of a blood vessel segment. According to the fuzzy mesh resolution, the total segment surface exchange area is evenly divided amongst the mass transfer mesh elements.

*Solution algorithms and Implementation*. In summary, the system to be solved is summarized in systems (1)–(2) for blood flow and hematocrit; and (3) for oxygen concentration in vasculature and tissue. Equation generation was written with object-oriented codes. All linear algebraic computations were performed in PETSc using the GMRES solver and a block Jacobi preconditioner [54,55]. We did not employ an algebraic multigrid solution algorithm.

## Supporting information

S1 Text Implementation details. An in-depth description of how to programmatically implement the anatomical labeling and formulate the equations.
(DOCX)

S2 Text Oxygen prediction for a second microcirculatory network. An investigation of mesh independence and oxygen distribution with an additional dataset to complement the dataset offered in the main text.
(DOCX)

S3 Text Discretization of the differential equations. A detailed explanation of the spatial discretization scheme used in this study with an illustrative example.
(DOCX)

S4 Text Boundary effects in smaller networks. An example of the effects of boundary condition choices on simulation results.
(DOCX)

## References

[1] Peers C, Dallas ML, Boycott HE, Scragg JL, Pearson HA, Boyle JP. Hypoxia and Neurodegeneration. Ann N Y Acad Sci. 2009 10 1;1177[1]:169–77. Available from: https://nyaspubs.onlinelibrary.wiley.com/doi/abs/10.1111/j.1749-6632.2009.05026.x. 19845619 10.1111/j.1749-6632.2009.05026.x

[2] Peers C, Pearson HA, Boyle JP. Hypoxia and Alzheimer’s disease. Essays Biochem. 2007 8 10;43:153–64. Available from: http://essays.biochemistry.org/content/43/153. 10.1042/BSE0430153 17705799

[3] Bullitt E, Zeng D, Mortamet B, Ghosh A, Aylward SR, Lin W, et al The effects of healthy aging on intracerebral blood vessels visualized by magnetic resonance angiography. Neurobiol Aging. 2010;31[2]:290–300. 10.1016/j.neurobiolaging.2008.03.022 18471935 PMC2806428

[4] Desjardins M, Berti R, Lefebvre J, Dubeau S, Lesage F. Aging-related differences in cerebral capillary blood flow in anesthetized rats. Neurobiol Aging. 2014 8 1;35[8]:1947–55. Available from: http://www.sciencedirect.com/science/article/pii/S0197458014001584. 10.1016/j.neurobiolaging.2014.01.136 24612672

[5] Faber JE, Zhang H, Lassance-Soares RM, Prabhakar P, Najafi AH, Burnett MS, et al Aging causes collateral rarefaction and increased severity of ischemic injury in multiple tissues. Arterioscler Thromb Vasc Biol. 2011;31[8]:1748–1756. 10.1161/ATVBAHA.111.227314 21617137 PMC3141082

[6] Murugesan N, Demarest TG, Madri JA, Pachter JS. Brain regional angiogenic potential at the neurovascular unit during normal aging. Neurobiol Aging. 2012;33[5]:1004–e1. 10.1016/j.neurobiolaging.2011.09.022 22019053 PMC3266473

[7] Casey MA, Feldman ML. Aging in the rat medial nucleus of the trapezoid body. III. Alterations in capillaries. Neurobiol Aging. 1985;6[1]:39–46. 10.1016/0197-4580(85)90070-3 4000384

[8] Wilkinson J, Hopewell J, Reinhold H. A quantitative study of age-related changes in the vascular architecture of the rat cerebral cortex. Neuropathol Appl Neurobiol. 1981;7[6]:451–462. 10.1111/j.1365-2990.1981.tb00245.x 7329517

[9] Silasi G, She J, Boyd JD, Xue S, Murphy TH. A Mouse Model of Small-Vessel Disease that Produces Brain-Wide-Identified Microocclusions and Regionally Selective Neuronal Injury. J Cereb Blood Flow Metab. 2015 5 1;35[5]:734–8. Available from: 10.1038/jcbfm.2015.8. 25690472 PMC4420872

[10] Xu X, Wang B, Ren C, Hu J, Greenberg DA, Chen T, et al Age-related Impairment of Vascular Structure and Functions. Aging Dis. 2017 10 1;8[5]:590–610. 10.14336/AD.2017.0430 28966804 PMC5614324

[11] Wang M, Iliff JJ, Liao Y, Chen MJ, Shinseki MS, Venkataraman A, et al Cognitive Deficits and Delayed Neuronal Loss in a Mouse Model of Multiple Microinfarcts. J Neurosci. 2012 12 12;32[50]:17948 10.1523/JNEUROSCI.1860-12.2012 23238711 PMC3541041

[12] Okamoto Y, Yamamoto T, Kalaria RN, Senzaki H, Maki T, Hase Y, et al Cerebral hypoperfusion accelerates cerebral amyloid angiopathy and promotes cortical microinfarcts. Acta Neuropathol (Berl). 2012 3 1;123[3]:381–94. Available from: https://link.springer.com/article/10.1007/s00401-011-0925-9. 10.1007/s00401-011-0925-9 22170742 PMC3282897

[13] Moeini M, Lu X, Avti PK, Damseh R, Bélanger S, Picard F, et al Compromised microvascular oxygen delivery increases brain tissue vulnerability with age. Sci Rep. 2018 5 29;8[1]:8219 Available from: https://www.nature.com/articles/s41598-018-26543-w. 10.1038/s41598-018-26543-w 29844478 PMC5974237

[14] Gagnon L, Sakadžić S, Lesage F, Musacchia JJ, Lefebvre J, Fang Q, et al Quantifying the Microvascular Origin of BOLD-fMRI from First Principles with Two-Photon Microscopy and an Oxygen-Sensitive Nanoprobe. J Neurosci. 2015 2 25;35[8]:3663–75. Available from: http://www.jneurosci.org/content/35/8/3663. 10.1523/JNEUROSCI.3555-14.2015 25716864 PMC4339366

[15] Lorthois S, Cassot F, Lauwers F. Simulation study of brain blood flow regulation by intra-cortical arterioles in an anatomically accurate large human vascular network. Part II: Flow variations induced by global or localized modifications of arteriolar diameters. NeuroImage. 2011 2 14;54[4]:2840–53. Available from: http://www.sciencedirect.com/science/article/pii/S1053811910013327. 10.1016/j.neuroimage.2010.10.040 21047557

[16] Kim JH, Ress D. Arterial impulse model for the BOLD response to brief neural activation. NeuroImage. 2016 1 1;124:394–408. Available from: http://www.sciencedirect.com/science/article/pii/S1053811915007892. 10.1016/j.neuroimage.2015.08.068 26363350 PMC4651785

[17] Griffeth VEM, Buxton RB. A theoretical framework for estimating cerebral oxygen metabolism changes using the calibrated-BOLD method: Modeling the effects of blood volume distribution, hematocrit, oxygen extraction fraction, and tissue signal properties on the BOLD signal. NeuroImage. 2011 9 1;58[1]:198–212. Available from: http://www.sciencedirect.com/science/article/pii/S1053811911006033. 10.1016/j.neuroimage.2011.05.077 21669292 PMC3187858

[18] Boas DA, Jones SR, Devor A, Huppert TJ, Dale AM. A vascular anatomical network model of the spatio-temporal response to brain activation. NeuroImage. 2008 4 15;40[3]:1116–29. Available from: http://www.sciencedirect.com/science/article/pii/S1053811908000086. 10.1016/j.neuroimage.2007.12.061 18289880 PMC2577617

[19] Zhong J, Kennan RP, Fulbright RK, Gore JC. Quantification of intravascular and extravascular contributions to BOLD effects induced by alteration in oxygenation or intravascular contrast agents. Magn Reson Med. 1998 10 1;40[4]:526–36. Available from: https://onlinelibrary.wiley.com/doi/abs/10.1002/mrm.1910400405. 9771569 10.1002/mrm.1910400405

[20] Sweeney PW, Walker-Samuel S, Shipley RJ. Insights into cerebral haemodynamics and oxygenation utilising in vivo mural cell imaging and mathematical modelling. Sci Rep. 2018 1 22;8[1]:1373 Available from: https://www.nature.com/articles/s41598-017-19086-z. 10.1038/s41598-017-19086-z 29358701 PMC5778006

[21] Lu H, Golay X, Pekar JJ, Zijl PCM van. Functional magnetic resonance imaging based on changes in vascular space occupancy. Magn Reson Med. 2003 8 1;50[2]:263–74. Available from: https://onlinelibrary.wiley.com/doi/abs/10.1002/mrm.10519. 12876702 10.1002/mrm.10519

[22] Hernández-Torres E, Kassner N, Forkert ND, Wei L, Wiggermann V, Daemen M, et al Anisotropic cerebral vascular architecture causes orientation dependency in cerebral blood flow and volume measured with dynamic susceptibility contrast magnetic resonance imaging. J Cereb Blood Flow Metab. 2017 3 1;37[3]:1108–19. Available from: 10.1177/0271678X16653134 27259344 PMC5363485

[23] Boxerman JL, Bandettini PA, Kwong KK, Baker JR, Davis TL, Rosen BR, et al The intravascular contribution to fmri signal change: monte carlo modeling and diffusion-weighted studies in vivo. Magn Reson Med. 1995 7 1;34[1]:4–10. Available from: https://onlinelibrary.wiley.com/doi/abs/10.1002/mrm.1910340103. 7674897 10.1002/mrm.1910340103

[24] Markuerkiaga I, Barth M, Norris DG. A cortical vascular model for examining the specificity of the laminar BOLD signal. NeuroImage. 2016 5 15;132:491–8. Available from: http://www.sciencedirect.com/science/article/pii/S1053811916001919. 10.1016/j.neuroimage.2016.02.073 26952195

[25] Levin JM, Frederick B deB, Ross MH, Fox JF, von Rosenberg HL, Kaufman MJ, et al Influence of baseline hematocrit and hemodilution on BOLD fMRI activation. Magn Reson Imaging. 2001 10 1;19[8]:1055–62. Available from: http://www.sciencedirect.com/science/article/pii/S0730725X0100460X. 10.1016/s0730-725x(01)00460-x 11711229

[26] Gould IG, Linninger AA. Hematocrit Distribution and Tissue Oxygenation in Large Microcirculatory Networks. Microcirculation. 2015 1 1;22[1]:1–18. Available from: http://onlinelibrary.wiley.com/doi/10.1111/micc.12156/abstract. 25040825 10.1111/micc.12156

[27] Gould IG, Tsai P, Kleinfeld D, Linninger A. The capillary bed offers the largest hemodynamic resistance to the cortical blood supply. J Cereb Blood Flow Metab. 2017 1 1;37[1]:52–68. Available from: 10.1177/0271678X16671146. 27780904 PMC5363755

[28] Gagnon L, Smith AF, Boas DA, Devor A, Secomb TW, Sakadžić S. Modeling of Cerebral Oxygen Transport Based on In vivo Microscopic Imaging of Microvascular Network Structure, Blood Flow, and Oxygenation. Front Comput Neurosci. 2016;10 Available from: https://www.frontiersin.org/articles/10.3389/fncom.2016.00082/full. 10.3389/fncom.2016.00082 PMC5006088 27630556

[29] Gagnon L, Sakadžić S, Lesage F, Mandeville ET, Fang Q, Yaseen MA, et al Multimodal reconstruction of microvascular-flow distributions using combined two-photon microscopy and Doppler optical coherence tomography. Neurophotonics. 2015 3;2[1]:015008 10.1117/1.NPh.2.1.015008 26157987 PMC4478873

[30] Fang Q, Sakadžić S, Ruvinskaya L, Devor A, Dale AM, Boas DA. Oxygen Advection and Diffusion in a Three Dimensional Vascular Anatomical Network. Opt Express. 2008 10 27;16[22]:17530–41. 10.1364/oe.16.17530 18958033 PMC2584207

[31] Secomb TW, Hsu R, Park EYH, Dewhirst MW. Green’s Function Methods for Analysis of Oxygen Delivery to Tissue by Microvascular Networks. Ann Biomed Eng. 2004 11 1;32[11]:1519–29. 10.1114/b:abme.0000049036.08817.44 15636112

[32] Hsu R, Secomb TW. A Green’s function method for analysis of oxygen delivery to tissue by microvascular networks. Math Biosci. 1989 9 1;96[1]:61–78. 10.1016/0025-5564(89)90083-7 2520192

[33] D’Angelo C. Finite element approximation of elliptic problems with Dirac measure terms in weighted spaces: applications to one-and three-dimensional coupled problems. SIAM J Numer Anal. 2012;50[1]:194–215.

[34] D’Angelo C, Quarteroni A. On the coupling of 1d and 3d diffusion-reaction equations: application to tissue perfusion problems. Math Models Methods Appl Sci. 2008;18[8]:1481–1504.

[35] Gjerde IG, Kumar K, Nordbotten JM, Wohlmuth B. Splitting method for elliptic equations with line sources. Comput Geosci. 2019;1715–39.

[36] Linninger AA, Gould IG, Marinnan T, Hsu C-Y, Chojecki M, Alaraj A. Cerebral Microcirculation and Oxygen Tension in the Human Secondary Cortex. Ann Biomed Eng. 2013 11 1;41[11]:2264–84. 10.1007/s10439-013-0828-0 23842693 PMC3878164

[37] Holter KE, Kehlet B, Devor A, Sejnowski TJ, Dale AM, Omholt SW, et al Interstitial solute transport in 3D reconstructed neuropil occurs by diffusion rather than bulk flow. Proc Natl Acad Sci. 2017 9 12;114[37]:9894–9. 10.1073/pnas.1706942114 28847942 PMC5604020

[38] Geuzaine C, Remacle J-F. Gmsh: A 3-D finite element mesh generator with built-in pre-and post-processing facilities. Int J Numer Methods Eng. 2009;79[11]:1309–1331.

[39] Briggs WL, McCormick SF, others. A multigrid tutorial. Vol. 72 Siam; 2000.

[40] Cattaneo L, Zunino P. A computational model of drug delivery through microcirculation to compare different tumor treatments. Int J Numer Methods Biomed Eng. 2014;30[11]:1347–1371. 10.1002/cnm.2661 25044965

[41] Notaro D, Cattaneo L, Formaggia L, Scotti A, Zunino P. A mixed finite element method for modeling the fluid exchange between microcirculation and tissue interstitium In: Advances in Discretization Methods. Springer; 2016 p. 3–25.

[42] Possenti L, di Gregorio S, Gerosa FM, Raimondi G, Casagrande G, Costantino ML, et al A computational model for microcirculation including Fahraeus-Lindqvist effect, plasma skimming and fluid exchange with the tissue interstitium. Int J Numer Methods Biomed Eng. 2019;35[3]. 10.1002/cnm.3165 30358172

[43] Possenti L, Casagrande G, Di Gregorio S, Zunino P, Costantino ML. Numerical simulations of the microvascular fluid balance with a non-linear model of the lymphatic system. Microvasc Res. 2019;122:101–110. 10.1016/j.mvr.2018.11.003 30448400

[44] Balogh P, Bagchi P. A computational approach to modeling cellular-scale blood flow in complex geometry. J Comput Phys. 2017 4 1;334:280–307.

[45] Balogh P, Bagchi P. Direct Numerical Simulation of Cellular-Scale Blood Flow in 3D Microvascular Networks. Biophys J. 2017 12 19;113[12]:2815–26. 10.1016/j.bpj.2017.10.020 29262374 PMC5770972

[46] Peyrounette M, Davit Y, Quintard M, Lorthois S. Multiscale modelling of blood flow in cerebral microcirculation: Details at capillary scale control accuracy at the level of the cortex. PLOS ONE. 2018 1 11;13[1]:e0189474 10.1371/journal.pone.0189474 29324784 PMC5764267

[47] El-Bouri WK, Payne SJ. Multi-scale homogenization of blood flow in 3-dimensional human cerebral microvascular networks. J Theor Biol. 2015 9 7;380:40–7. 10.1016/j.jtbi.2015.05.011 25986433

[48] Ghaffari M, Tangen K, Alaraj A, Du X, Charbel FT, Linninger AA. Large-scale subject-specific cerebral arterial tree modeling using automated parametric mesh generation for blood flow simulation. Comput Biol Med. 2017 12 1;91:353–65. 10.1016/j.compbiomed.2017.10.028 29126049 PMC5696053

[49] Ghaffari M, Alaraj A, Du X, Zhou XJ, Charbel FT, Linninger AA. Quantification of near-wall hemodynamic risk factors in large-scale cerebral arterial trees. Int J Numer Methods Biomed Eng. 2018;34[7]. 10.1002/cnm.2987 29601146 PMC6043404

[50] Ghaffari M, Hsu C-Y, Linninger AA. Automatic reconstruction and generation of structured hexahedral mesh for non-planar bifurcations in vascular networks In: Computer Aided Chemical Engineering. Elsevier; 2015 p. 635–640.

[51] Kim JH, Astary GW, Chen X, Mareci TH, Sarntinoranont M. Voxelized model of interstitial transport in the rat spinal cord following direct infusion into white matter. J Biomech Eng. 2009;131[7]. 10.1115/1.3169248 19640132 PMC2906454

[52] Hartung G, Vesel C, Morley R, Alaraj A, Sled J, Kleinfeld D, et al Simulations of blood as a suspension predicts a depth dependent hematocrit in the circulation throughout the cerebral cortex. PLOS Comput Biol. 2018 11 19;14[11]. 10.1371/journal.pcbi.1006549 30452440 PMC6277127

[53] Blinder P, Tsai PS, Kaufhold JP, Knutsen PM, Suhl H, Kleinfeld D. The cortical angiome: an interconnected vascular network with noncolumnar patterns of blood flow. Nat Neurosci. 2013 7;16[7]:889–97. 10.1038/nn.3426 23749145 PMC4141079

[54] Balay S, Abhyankar S, Adams MF, Brown J, Brune P, Buschelman K, et al PETSc Web page [Internet]. 2019 Available from: https://www.mcs.anl.gov/petsc.

[55] Balay S, Abhyankar S, Adams MF, Brown J, Brune P, Buschelman K, et al PETSc Users Manual [Internet]. Argonne National Laboratory; 2019 Report No.: ANL-95/11-Revision 3.11. Available from: https://www.mcs.anl.gov/petsc.

[56] Mintun MA, Lundstrom BN, Snyder AZ, Vlassenko AG, Shulman GL, Raichle ME. Blood flow and oxygen delivery to human brain during functional activity: theoretical modeling and experimental data. PNAS. 2001;98[12]:6859–64. 10.1073/pnas.111164398 11381119 PMC34443

[57] Truskey G, Yuan F, Katz D. Transport Phenomena in Biological Systems. Pearson Prentice Hall;

[58] Gould IG, Linninger AA. Hematocrit Distribution and Tissue Oxygenation in Large Microcirculatory Networks. Microcirculation. 2015 1 1;22[1]:1–18. 10.1111/micc.12156 25040825

[59] Linninger A, Hartung G, Badr S, Morley R. Mathematical synthesis of the cortical circulation for the whole mouse brain-part I: theory and image integration. Comput Biol Med. 2019; 10.1016/j.compbiomed.2019.05.004 31247510

[60] Lorthois S, Cassot F, Lauwers F. Simulation study of brain blood flow regulation by intra-cortical arterioles in an anatomically accurate large human vascular network: Part I: methodology and baseline flow. NeuroImage. 2011 1 15;54[2]:1031–42. 10.1016/j.neuroimage.2010.09.032 20869450

[61] Schmid F, Tsai PS, Kleinfeld D, Jenny P, Weber B. Depth-dependent flow and pressure characteristics in cortical microvascular networks. PLOS Comput Biol. 2017 2 14;13[2]. 10.1371/journal.pcbi.1005392 28196095 PMC5347440

[62] Nicolaides RA. Deflation of conjugate gradients with applications to boundary value problems. SIAM J Numer Anal. 1987;24[2]:355–365.

[63] Saad Y. Iterative methods for sparse linear systems. Vol. 82 siam; 2003.

[64] May DA, Sanan P, Rupp K, Knepley MG, Smith BF. Extreme-scale multigrid components within PETSc. In: Proceedings of the Platform for Advanced Scientific Computing Conference. 2016. p. 1–12.

[65] Stüben K. A review of algebraic multigrid In: Numerical Analysis: Historical Developments in the 20th Century. Elsevier; 2001 p. 331–359.

[66] Hartung G. Multi-Scale Simulation of Cerebral Blood Flow and Oxygen Exchange for the Entire Mouse Brain. University of Illinois at Chicago; 2020.

[67] Erbertseder K, Reichold J, Flemisch B, Jenny P, Helmig R. A coupled discrete/continuum model for describing cancer-therapeutic transport in the lung. PLoS One. 2012;7[3]. 10.1371/journal.pone.0031966 22438873 PMC3305605

[68] Koch T, Schneider M, Helmig R, Jenny P. Modeling tissue perfusion in terms of 1d-3d embedded mixed-dimension coupled problems with distributed sources. J Comput Phys. 2020;410.

[69] Noble RW, Parkhurst LJ, Gibson QH. The effect of pH on the reactions of oxygen and carbon monoxide with the hemoglobin of the carp, Cyprinus carpio. J Biol Chem. 1970;245[24]:6628–6633. 5482773

[70] Tan AL, De Young A, Noble RW. The pH dependence of the affinity, kinetics, and cooperativity of ligand binding to carp hemoglobin, Cyprinus carpio. J Biol Chem. 1972;247[8]:2493–2498. 5019960

[71] Tsai PS, Kaufhold JP, Blinder P, Friedman B, Drew PJ, Karten HJ, et al Correlations of Neuronal and Microvascular Densities in Murine Cortex Revealed by Direct Counting and Colocalization of Nuclei and Vessels. J Neurosci. 2009 11 18;29[46]:14553–70. 10.1523/JNEUROSCI.3287-09.2009 19923289 PMC4972024

[72] Shih AY, Driscoll JD, Drew PJ, Nishimura N, Schaffer CB, Kleinfeld D. Two-Photon Microscopy as a Tool to Study Blood Flow and Neurovascular Coupling in the Rodent Brain. J Cereb Blood Flow Metab. 2012 7 1;32[7]:1277–309. 10.1038/jcbfm.2011.196 22293983 PMC3390800

[73] Kaufhold JP, Tsai PS, Blinder P, Kleinfeld D. Vectorization of optically sectioned brain microvasculature: Learning aids completion of vascular graphs by connecting gaps and deleting open-ended segments. Med Image Anal. 2012 8;16[6]:1241–58. 10.1016/j.media.2012.06.004 22854035 PMC3443315

[74] Finikova OS, Lebedev AY, Aprelev A, Troxler T, Gao F, Garnacho C, et al Oxygen microscopy by two-photon-excited phosphorescence. ChemPhysChem. 2008;9[12]:1673–1679. 10.1002/cphc.200800296 18663708 PMC2645351

[75] Tellegen BDH. A general network theorem, with applications. Philips Res Rept. 1952;7:259–69.

