# Supplementary material for: Voxelized simulation of cerebral oxygen perfusion elucidates hypoxia in aged mouse cortex
Source: PLoS Comput Biol. 2021 Jan 28;17(1):e1008584. doi: 10.1371/journal.pcbi.1008584 (PMC7842915; doi:10.1371/journal.pcbi.1008584)
Supplement: S1 Text — An in-depth description of how to programmatically implement the anatomical labeling and formulate the equations. (DOCX) [file pcbi.1008584.s001.docx]

# S1 Supplement. implementation details

The act of labeling a 3D Cartesian (cuboid) structure is necessary prior to equation generation. One common problem with mesh instability at finer resolutions is created if the network edge lengths remain fixed as the mesh density increases (=edge length decreases in mesh). The best results for simulations occur when the characteristic edge length of the mesh and the vascular network are commensurate as previously noted (30,36). Here, we describe a new method for how to efficiently label an entire cuboid mesh using a dense vascular structure. To ensure numerical stability with increasing mesh density, vascular segment partitioning prior to labeling ensures the characteristic edge length of the vascular network compatible with that of the mesh. Once the vasculature is adequately refined, the efficient mesh labeling procedure cycles through all the vascular elements to track all connectivity between the mesh and the network. More details on vascular partitioning and mesh labeling are provided in the next sections.

*Vascular partitioning.* In order to ensure similar characteristic length scales for the Cartesian mesh and vascular network, we propose a method for partitioning the vascular network dynamically (subdividing the segments). Each vessel in the vascular network must pass a criterion that ensures that no vessel spans more than 2 adjacent cells. In other words, the mesh cell indices (i, j, and k positions in the x, y, and z directions respectively) of the endpoints must not differ by more than 1. Segments that fail are cut in half. Note, after a segment is cut in half, both sub-segments must be evaluated against the criteria test again.

We verified the vascular connectivity was preserved during simulation by calculating the flux balance across each vascular node. We ensure these mass balances independent of the equation generation/solving stages by recalculating all fluxes between adjacent volumes in the system and then calculating mass balance error from the sum of fluxes. We then ensure that the divergence of all balances is less than the preset tolerance. In the event our method did not represent the vascular connectivity correctly, the mass balances would exceed the tolerance during this check phase. We also ensure that the segment-to-segment and segment-tissue connectivity is preserved during the oxygen simulation stage by performing another flux balance check at this stage again.

*Mesh labeling using vascular network*. The next step entails labeling each cell into one of three categories: intravascular (blood, red), endothelial layer (grey), and extravascular space (blue). The method takes advantage of the Cartesian coordinate system to avoid having to search neighborhood connectivity that would be required in unstructured, tetrahedral meshes. In this way, the algorithm drastically reduces the number of floating point operations required for labeling. Specifically, the program cycles through all vessels in the vascular network. The bounding box of each vessel is computed and translated to dimensional indices (i, j, and k positions in the cuboid mesh). The maximum and minimum indices in each dimension characterize the new search domain for the cylinder. Each mesh cell within the subdomain is then compared to the cylinder and labeled as intravascular, endothelial, or extravascular. A pseudocode for these steps is offered in Table A in S1 Text.

The radial distance (=perpendicular distance) between the center of each subdomain mesh element and the vascular segment is then computed. If the axial distance is determined to be between the first and second point, axially, then the radial distance between the cell and the segment characterizes whether or not the cell is within the cylinder (if distance < radius, the mesh cell is within the cylinder). Due to the gaps between adjacent segments modeled as perfect cylinders, the ends of the cylinder are considered spherical with equivalent radii to the parent cylinder.

Identifying whether a cell center lies in a cylinder or not can be performed with vector operations. This efficient algorithm simply characterizes the vessel centerline as a vector and uses sine and cosine operations to evaluate the radial and axial distance, respectively.

Table A. Pseudocode for labeling a mesh with a dense vascular structure

| 1. | FUNCTION Label3DMesh(mesh,nwk,w) |
| --- | --- |
| 2. | FOR iFace = 1 TO nwk.nFaces DO |
| 3. | getPointsForFace(iFace, p1Idx, p2Idx); |
| 4. | dia = nwk.dia[iFace]; |
| 5. | [minI,minJ,minK,maxI,maxJ,maxK]=findBoundingBoxOfFace(p1,p2, radius); |
| 6. | FOR i = minI TO maxI DO |
| 7. | FOR j = minJ TO maxJ DO |
| 8. | FOR k = minK TO maxK DO |
| 9. | cellIdx = mesh.getGlobalIdxFromIJK(i,j,k); |
| 10. | cellCenter = mesh.getCellCenter(cellIdx); |
| 11. | result[cellIdx]=…  labelCellWithCylinder(p1,p2,cellCenter,dia/2,w); |
| 12. | ENDFOR |
| 13. | ENDFOR |
| 14. | ENDFOR |
| 15. | ENDFOR |

The endothelial can be characterized by the radius of the vessel $\pm w/2$, orhalf the wall thickness of the endothelial layer. The radial distance and subsequent labeling between a new point (cell center) for a segment centerline and cell center is given by the following pseudocode. In our actual implementation, we also expand each cylindrical segment with a hemisphere protruding outwards from each of the two terminal nodes, but this additional function is somewhat geometrically involved. We therefore chose to omit it from Table B in S1 Text for better clarity of the overall idea.

Table B. Pseudocode for labeling a given cell in reference to a single vascular cylindrical segment

| 1. | FUNCTION labelCellWithCylinder (p1,p2,cellCenter,r,w):integer; |
| --- | --- |
| 2. | v1=getAsVector(p1,p2); v2=getAsVector(p1,cellCenter); |
| 3. | c=dot(v1,v2)/(norm(v1)*norm(v2)); H=norm(v2); |
| 4. | d=H*power(1-c*c,0.5); |
| 5. | IF (d < r – w/2) THEN label=endothelium; |
| 6. | ELSEIF (d < r + w/2) THEN label=endothelium; |
| 7. | ELSEIF (d < r) THEN label=interior; |
| 8. | ELSE THEN label=extravascular; |
| 9. | RETURN label; |

The variable $d$ represents the perpendicular distance between the mesh cell center and the vascular centerline. This can be calculated with the help of defining the centerline and cell center in vector form. The scaled sine of the angle between these vectors is perpendicular distance from the centerline:

| $d=\left\vert v_{2} \right\vert\left( 1-\left[ \frac{v_{1}\cdot v_{2}^{T}}{\left\vert v_{1} \right\vert\cdot\left\vert v_{2} \right\vert} \right]^{2} \right)^{1/2}$ | (S1.1) |
| --- | --- |

Where $v_{1}$ is the vector connecting the segment endpoints (=vessel centerline). The vector $v_{2}$ indicates the vector between the first endpoint and the mesh cell center.

*Hierarchy of labeling.* At bifurcations and segments with extreme curvature, two or more subsequent segments may overlap. As a consequence, some mesh cells located in the overlapping domain may be subject to labeling from more than one vascular segments. In order to avoid ambiguous labeling, a hierarchical labeling logic has been implemented to determine a unique cell label. The logic has three rules: (i) An endothelial label always overrides an extravascular label. (ii) Intravascular label always overrides extravascular *and* endothelial labels. (iii) Finally, for cells with the same hierarchy, the upstream segment label has precedence. Examples of this labeling are given in the Fig in S1 Text, frame D. Examples of labeling without competition are shown in the Fig in S1 Text, frames A-C.

| 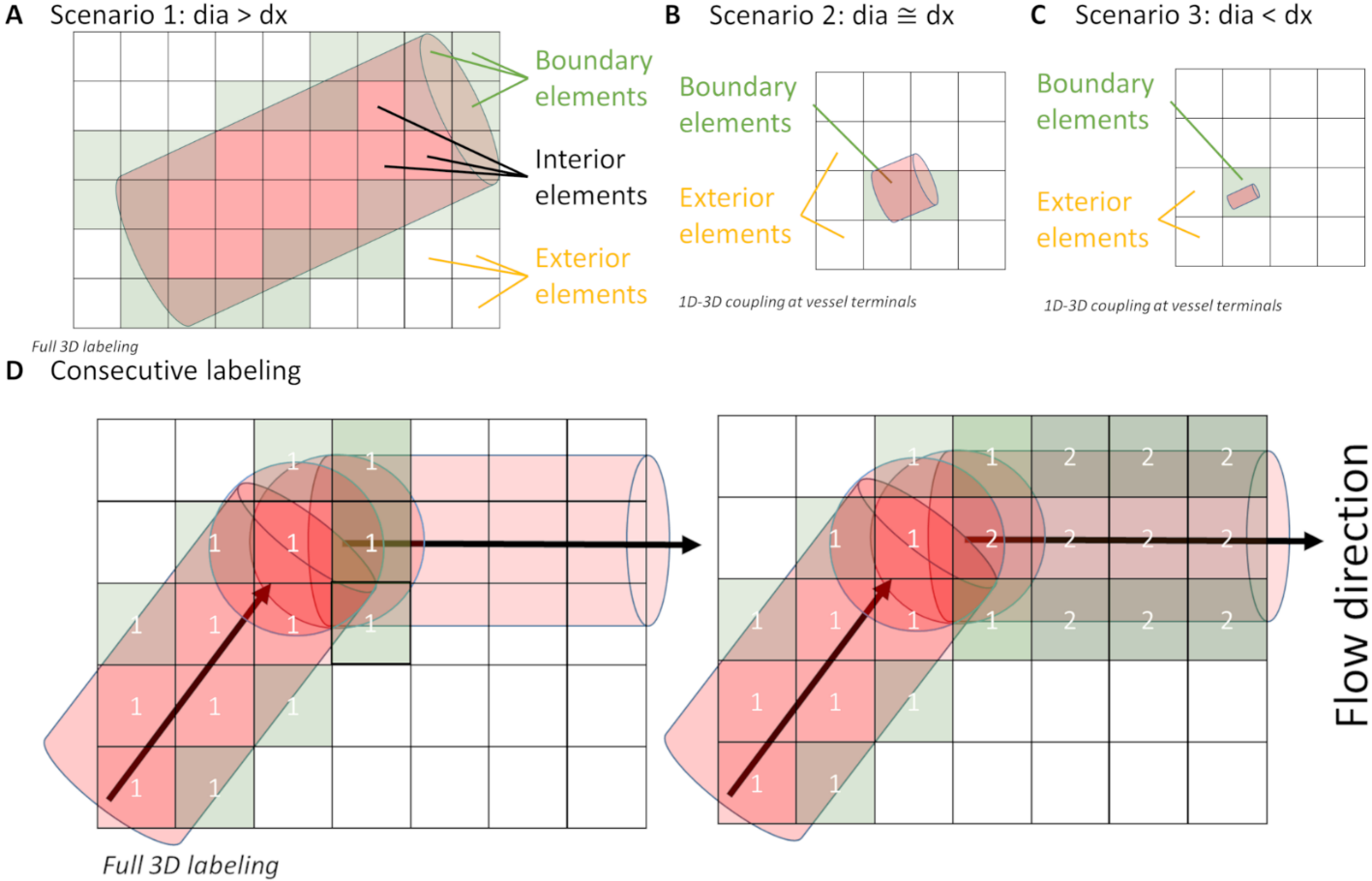 |
| --- |
| S1. Fig1. Examples of mesh labeling for blood vessels of different length scales. A) A large vessel with large diameter (dia) compared to characteristic mesh edge length (dx) may label many interior mesh elements and a concise endothelial layer (note, this vessel does not reflect partitioning). Colors indicate interior (red), boundary (green) and extravascular (exterior-white). B) A vessel that has a similar width to a single mesh element will merely label the element as endothelial elements and exhibit mass transfer to it. C) Smaller vascular segments may be entirely contained within a single mesh element. D) The progression of labeling as two adjacent segments are labeled. The number inside each mesh cell indicates which vascular element the cell is connected with. |

In addition, for all edge and the interior cells, the index of the associated segment is stored in the connectivity incidence matrices, where each row of the *cell_to_segment* connectivity matrix stores which vascular segments connecting to the cell, and the *segment_to_cell* connectivity matrix stores the cell indices concerted to a vascular segment.

*Automatic equation generation.* For tissue elements, numerical expressions for the face fluxes for each cuboid cell are built by one of three methods: (i) *Diffusion flux* equations are created between two cells belonging to the same group (extravascular elements, endothelial elements). Moreover, diffusion occurs from the endothelial surface to the extravascular tissue space(=entirs of $M_{d}$. (ii) *Mass transfer flux* equations are generated between an endothelial element and the corresponding vascular node (entries in $C_{3}$). (iii) Flux equations are omitted between all intravascular elements. Instead, the equations to determine the concentration for intravascular mesh elements, $c_{t}^{j}$ (=red cuboid), is obtained by equating it to the concentration of the corresponding network vascular node, $c_{v}^{i}$, as indicated in (S1.2)

| $c_{t}^{j}=c_{v}^{i}$ | (S1.2) |
| --- | --- |

The mass transfer matrices $C_{3}$ can be automatically populated with the help of the *cell_to_segment* and *segment_to_cell* data structures (=integer connectivity matrices). Specifically, each *i*^th^ node in the vascular network generates a contribution to the *j*^th^ element corresponding to the tissue node. These matrices can be filled by cycling through all nodes of the vascular structure.

For clarity, we revisit the final master equation for coupled oxygen transport between the vasculature and the tissue mesh, system (3) in the main manuscript. Here, we expand the unknonw vectors of the tissue mesh in more detail to more clearly define the implementation of the flux equations with the help of the mesh masking labels (intravascular, endothelial, and extravascular volumes). The coupled equations can then be expressed in terms of four independent vascular network and tissue variable sets: The oxygen concentration on the vascular nodes, $c_{v}$, the tissue endothelial elements, $c_{t}^{e}$, the extravascular tissue elements, $c_{t}$, and the intravascular tissue elements, $c_{t}^{v}$. These endothelial and the tissue nodes inside the Cartesian mesh were lumped in the main manuscript in (3). Moreover, the intraluminal nodes were eliminated with the help of the equalities in (S1.2). Alternatively you could solve for these equations with the help of the fifth matrix in (S1.3).

| $\left( \left[ \begin{matrix} M_{c}\left( f,h \right) & \begin{matrix} 0 & 0 & 0 \end{matrix} \\ \begin{matrix} 0 \\ 0 \\ 0 \end{matrix} & \begin{matrix} 0 & 0 & 0 \\ 0 & 0 & 0 \\ 0 & 0 & 0 \end{matrix} \end{matrix} \right]-\left[ \begin{matrix} C_{3}^{T}G_{1}{C_{3}} \\ 0 \\ 0 \end{matrix} \right]+\left[ \begin{matrix} 0 & \begin{matrix} 0 & 0 \end{matrix} & 0 \\ \begin{matrix} 0 \\ 0 \end{matrix} & M_{d} & \begin{matrix} 0 \\ 0 \end{matrix} \\ 0 & \begin{matrix} 0 & 0 \end{matrix} & 0 \end{matrix} \right]-\left[ \begin{matrix} 0 & \begin{matrix} 0 & 0 \end{matrix} & 0 \\ \begin{matrix} 0 \\ 0 \end{matrix} & R_{2} & \begin{matrix} 0 \\ 0 \end{matrix} \\ 0 & \begin{matrix} 0 & 0 \end{matrix} & 0 \end{matrix} \right]+\left[ \begin{matrix} 0 & \begin{matrix} 0 & 0 \end{matrix} & 0 \\ \begin{matrix} 0 \\ 0 \end{matrix} & \begin{matrix} 0 & 0 \\ 0 & 0 \end{matrix} & \begin{matrix} 0 \\ 0 \end{matrix} \\ -C_{4} & \begin{matrix} 0 & 0 \end{matrix} & I \end{matrix} \right] \right)\left[ \begin{aligned} c_{v} \\ c_{t}^{e} \\ c_{t} \\ c_{t}^{v} \end{aligned} \right]=\left[ \begin{aligned} D_{1}\bar{c_{v}} \\ 0 \\ D_{2}\bar{c_{t}} \\ 0 \end{aligned} \right]$ | (S1.3) |
| --- | --- |

Where oxygen tension in blood vessels, $c_{v}$, the extravascular mesh cells, $c_{t}$, the endothelial cells, $c_{t}^{e}$ and the intravascular cells, $c_{t}^{v}$ are solved for simultaneously.

*Description of submatrices.* Here, $M_{c}\in\mathfrak{R}^{nPts x nPts}$ is a matrix of convection of oxygen through the vascular network. The rectangular matrix $C_{3}\in\mathfrak{R}^{nPts x nPts+nVol}$ represents the connectivity between the vascular nodes and the corresponding endothelial mesh elements identified by the labeling algorithm. The diagonal matrix of mass transfer conductivity $G_{1}=\frac{UA}{w}$ is used to scale the coefficients of the mass transfer connectivity. The matrix $M_{d}\in\mathfrak{R}^{nVol x nVol}$ enforces the diffusion between endothelial and extravascular tissue mesh elements. The diagonal matrix $R_{2}\in\mathfrak{R}^{nVol x nVol}$ contains the reaction rate constants, $k_{met}$, and the cuboid volume ($V_{t}$). The equality constraints between vascular nodes and corresponding intravascular mesh cells in (S1.2) is enforced by the identity matrix and connectivity matrix $C_{4}$ according to a one-to-many association.

*Right hand side (RHS)*. $\bar{c_{v}}$ represents the boundary conditions for the vasculature and $D_{1}$ is the incidence matrix of the inlet boundary nodes in the vasculature.$\bar{c_{t}}$ represents the boundary conditions for the tissue which is similarly aided by the decision matrix $D_{2}$.
