# Supplementary material for: Voxelized simulation of cerebral oxygen perfusion elucidates hypoxia in aged mouse cortex
Source: PLoS Comput Biol. 2021 Jan 28;17(1):e1008584. doi: 10.1371/journal.pcbi.1008584 (PMC7842915; doi:10.1371/journal.pcbi.1008584)
Supplement: S2 Text — An investigation of mesh independence and oxygen distribution with an additional dataset to complement the dataset offered in the main text. (DOCX) [file pcbi.1008584.s002.docx]

# S2 Supplement. Oxygen prediction for a second microcirculatory network

In the main manuscript, we showed that the proposed mesh-free method was capable of resolving the microgradients around blood vessels for a large sample of the microcirculation in the mouse cortex. We further tested the performance of the new methodology on a second sample of a somatosensory from the Kleinfeld data set (E1.1).

The simulation results depicted in S2A and S2B show that simulations on the second structure were also successful in predicting reasonable oxygen tensions. S2A summarizes mesh independence using raytracing and should be compared to the main body Fig 3. S2B elucidates the sharp profile resolution using 2D contour maps with results comparable to main body Fig 4. We have further available in our lab two more empirical data structures and more than 80 synthetic data structures. The oxygen simulation converged on all of these data structures with similar performance than the two cases reported in this article. The expanded simulation results for these structures confirms robust performance of the methodology for arbitrary networks.

| 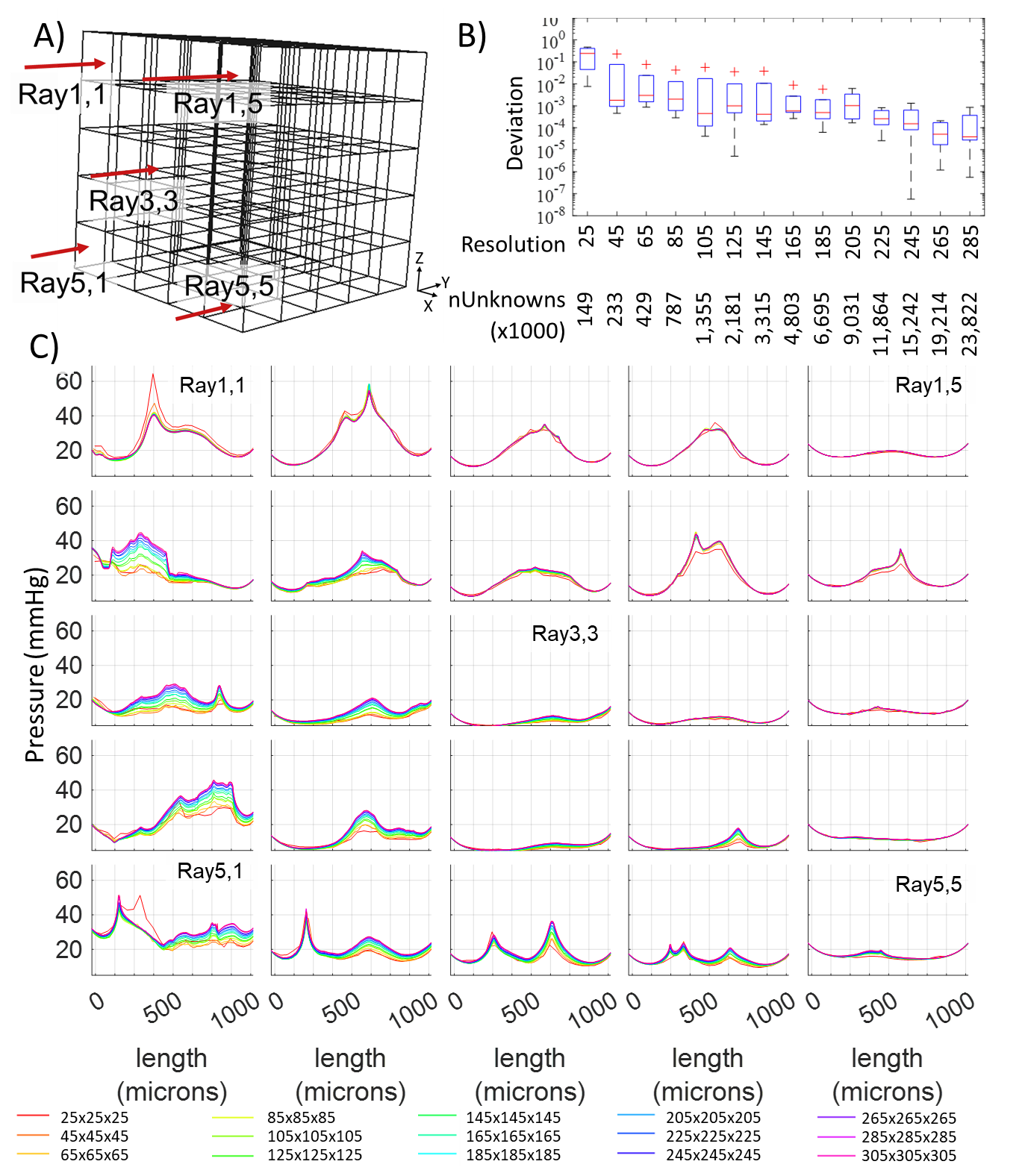 |
| --- |
| S2A. Detailed analysis of extravascular space in an oxygen simulation for a single empirical network (specimen E1.1). A) The 3D image shows the dispersion of oxygen originating in the vascular inlets and dispersing throughout the tissue domain. B) The convergence of values for many rays at y=718μm shows stable convergence and an acceptable tolerance at ~105 mesh elements per dimension. C) Raytraces through the domain exemplify the spatial complexity of oxygen tension. Deviation is calculated from the highest density mesh of resolution 305x305x305 volumes per dimension corresponding to ~29M total elements. |

| 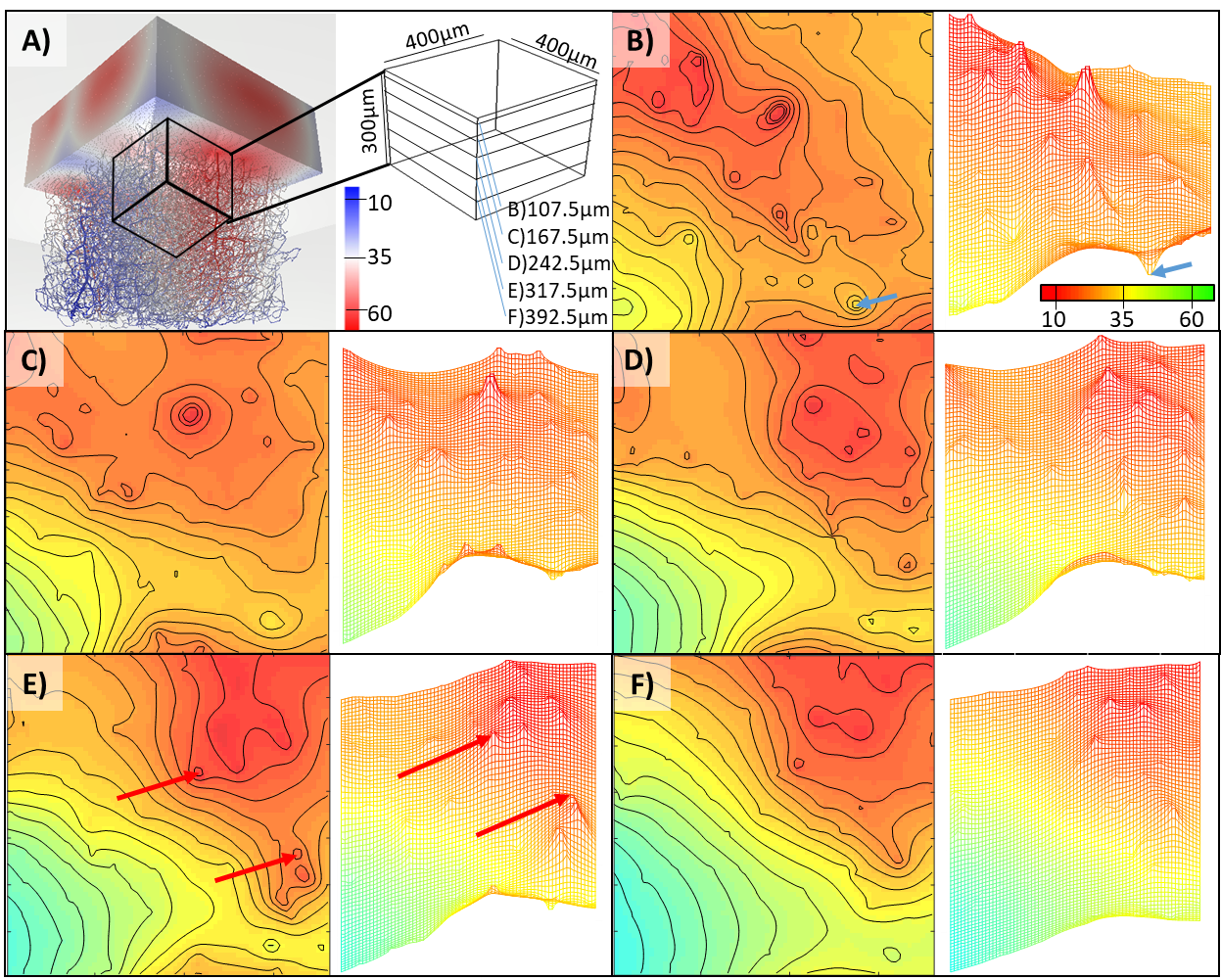 |
| --- |
| S2B. Oxygen predictions throughout the extravascular space in an empirically derived network specimen (E1.1). A) Visualization of a portion of the extravascular space with a block cutout defined by 5 observation planes similar to those experimentally imaged (107.5, 167.5, 242.5, 317.5, and 392.5μm below the cortical surface). B-F) The visualization of oxygen at different cortical layers shows peaks and steep microgradients surrounding main feeding (identified by red arrows) and draining (identified by blue arrows) vessels. |
