# Supplementary material for: Voxelized simulation of cerebral oxygen perfusion elucidates hypoxia in aged mouse cortex
Source: PLoS Comput Biol. 2021 Jan 28;17(1):e1008584. doi: 10.1371/journal.pcbi.1008584 (PMC7842915; doi:10.1371/journal.pcbi.1008584)
Supplement: S3 Text — A detailed explanation of the spatial discretization scheme used in this study with an illustrative example. (DOCX) [file pcbi.1008584.s003.docx]

# S3 Supplement. Discretization of the differential equations

As an example of the discretization scheme used in this manuscript, we will look at an extravascular volume element and the transport equations governing oxygen in that element (steady-state diffusion with 0^th^ order reactions). The analytic representation of the diffusion-reaction problem in steady state is presented in (S3.1) is in analytic form. On finite grids (or meshes), the equations must be discretized and applied to the elements of the grid.

| $-\frac{\partial}{\partial x}\left( -D\frac{\partial c}{\partial x} \right) =-R_{0}$ | (S3.1) |
| --- | --- |

To understand how to apply the transport equation from analytics to a discretized system, a decision must be made to either enforce a finite element method (FEM) or finite volume method (FVM). The FEM applies (S3.1) directly for each element, making it easier to implement, but the FEM suffers from discontinuities at non-identical interfaces (interfaces between two tissues for instance) at low mesh density. This occurs because the balance equations approaching the interface from one side to not match the balance equations as they approach from the other side. Physically, this can be interpreted from the balance equation; (S3.1) enforces that the second derivative of the state across a volume is equal to the reaction rate (in the steady-state case). To overcome discontinuities at boundary interfaces, a large number of grid points is required (high density mesh).

The FVM works on the integral form of the equation. Instead of solving for the acceleration at each node, the divergence is calculated as the sum of fluxes through the volume. This enforces the flux out of one element is arrives at the neighboring element which guarantees mass is conserved and reduces the occurrence of discontinuities at lower mesh densities. Because concentration must be conserved in these predictions, FVM is the better choice for discretization methods. To implement an FVM method, (S3.1) must be integrated as follows:

| $0=\int_{L}^{R} -\frac{\partial}{\partial x}\left( -D\frac{\partial c}{\partial x} \right) dx-\int_{L}^{R} R_{0} dx$ | (S3.2) |
| --- | --- |

Where R is right and L is left branch. When evaluated numerically:

| $0=\int_{L}^{R} -\frac{\partial}{\partial x}\left( D\frac{c_{i}-c_{i+1}}{x} \right) dx-\int_{L}^{R} R_{0} dx$ | (S3.3) |
| --- | --- |

Note that the second part of the fundamental theorem of calculus (also known as the Newton‑Leibniz axiom) gives:

| If  ${f(x)}^{'}=F(x)$  Then:  $\int_{L}^{R} F\left( x \right) dx=f\left( R \right)-f\left( L \right)$  Giving:  $\int_{L}^{R} f'\left( x \right) dx=f\left( R \right)-f\left( L \right)$ | (S3.4) |
| --- | --- |

Which is gives the integral form of the balance equation as:

| $D\frac{c_{i-1}-2c_{i}+c_{i+1}}{dx}=R_{0} dx$ | (S3.5) |
| --- | --- |

In application to a Cartesian 3D grid, (S3.1) can be expanded to:

| $\int_{S}^{D} \int_{B}^{T} \int_{L}^{R} R_{0} dxdydz=\int_{S}^{D} \int_{B}^{T} \int_{L}^{R} -\frac{\partial}{\partial x}\left( -D\frac{\partial c}{\partial x} \right)-\frac{\partial}{\partial y}\left( D\frac{\partial c}{\partial y} \right)-\frac{\partial}{\partial z}\left( D\frac{\partial c}{\partial z} \right) dx dydz$ | (S3.6) |
| --- | --- |

Where S is shallow and D is deep into the page. Evaluating the integral gives:

| $\int_{S}^{D} \int_{B}^{T} \int_{L}^{R} R_{0} dxdydz=\int_{S}^{D} \int_{B}^{T} \int_{L}^{R} -\frac{\partial}{\partial x}\left( -D\frac{\partial c}{\partial x} \right)-\frac{\partial}{\partial y}\left( D\frac{\partial c}{\partial y} \right)-\frac{\partial}{\partial z}\left( D\frac{\partial c}{\partial z} \right) dx dydz$  $=\int_{B}^{T} \int_{L}^{R} \left. D\frac{c_{i}-c_{i+1}}{\Delta x} \right\vert_{L}-\left. D\frac{c_{i}-c_{i+1}}{\Delta x} \right\vert_{R}-\frac{\partial}{\partial y}\left( D\frac{\partial c}{\partial y} \right)\Delta x -\frac{\partial}{\partial z}\left( D\frac{\partial c}{\partial z} \right)\Delta x dydz$  $=\int_{B}^{T} \left( \left. D\frac{c_{i}-c_{i+1}}{\Delta x} \right\vert_{L}-\left. D\frac{c_{i}-c_{i+1}}{\Delta x} \right\vert_{R} \right)\Delta y+\left( \left. D\frac{c_{i}-c_{i+1}}{\Delta y} \right\vert_{B}-\left. D\frac{c_{i}-c_{i+1}}{\Delta y} \right\vert_{T} \right)\Delta x -\frac{\partial}{\partial z}\left( D\frac{\partial c}{\partial z} \right)\Delta x\Delta y dz$  $R_{0}\Delta x\Delta y\Delta z=\left( \left. D\frac{c_{i}-c_{i+1}}{\Delta x} \right\vert_{L}-\left. D\frac{c_{i}-c_{i+1}}{\Delta x} \right\vert_{R} \right)\Delta y\Delta z+\left( \left. D\frac{c_{i}-c_{i+1}}{\Delta y} \right\vert_{B}-\left. D\frac{c_{i}-c_{i+1}}{\Delta y} \right\vert_{T} \right)\Delta x\Delta z+\left( \left. D\frac{c_{i}-c_{i+1}}{\Delta x} \right\vert_{S}-\left. D\frac{c_{i}-c_{i+1}}{\Delta x} \right\vert_{D} \right)\Delta x\Delta y$ | (S3.7) |
| --- | --- |

This final form is the diffusivity in each dimension multiplied by the cross sectional area perpendicular to the diffusive dimension. Moreover, the reaction is multiplied by the mesh element volume. Note, this formulation assumes isotropic, uniform diffusivity. This implementation is similar to that proposed by Patankar (77), specifically Sections 3.2.4 and 3.3.

A graphical example. An example mesh has been created in 3D and visualized in the Fig in S3 Text.

| 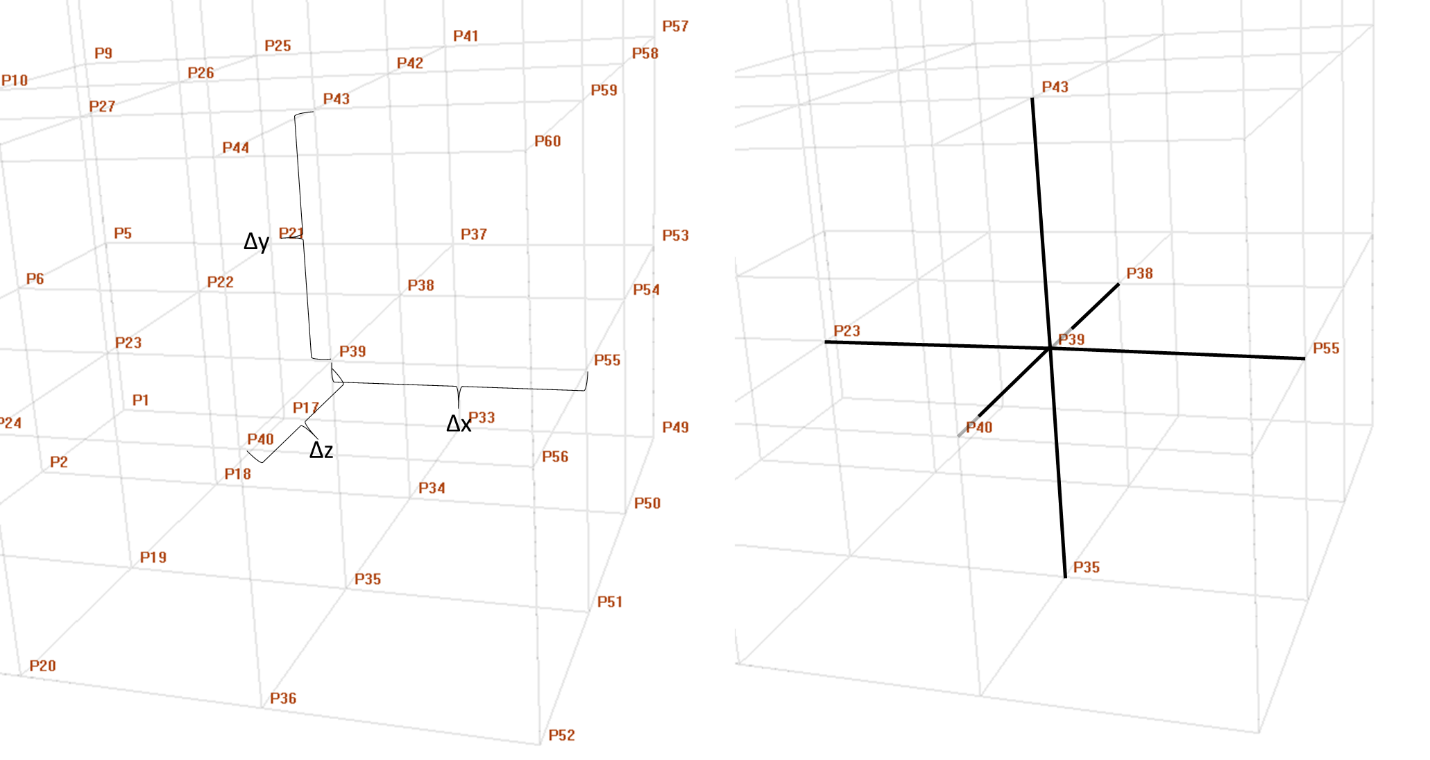 |
| --- |
| S3. Fig. 1-dimensional grid system on which the transport equations will be discretized. |

Evaluated at node 39 (assuming nodes are centers of hexahedral volumes and a diffusivity value of 1) gives:

| $R_{0}\Delta x\Delta y\Delta z=\frac{\Delta y\Delta z}{\Delta x}\left( c_{23}-2c_{39}+c_{55} \right)$  $+\frac{\Delta x\Delta z}{\Delta y}\left( c_{35}-2c_{39}+c_{38} \right)$ $+\frac{\Delta x\Delta y}{\Delta z}\left( c_{40}-2c_{39}+c_{38} \right)$ | (S3.8) |
| --- | --- |

Where the connected nodes to node 39 are 23 (L), 55 (R), 38 (D), 40 (S), 43 (T), and 35 (B). Where the grid is still evenly spaced and the values of flux are in moles/s.
