# Supplementary material for: Voxelized simulation of cerebral oxygen perfusion elucidates hypoxia in aged mouse cortex
Source: PLoS Comput Biol. 2021 Jan 28;17(1):e1008584. doi: 10.1371/journal.pcbi.1008584 (PMC7842915; doi:10.1371/journal.pcbi.1008584)
Supplement: S4 Text — An example of the effects of boundary condition choices on simulation results. (DOCX) [file pcbi.1008584.s004.docx]

# S4 Text. Boundary effects in smaller networks

| 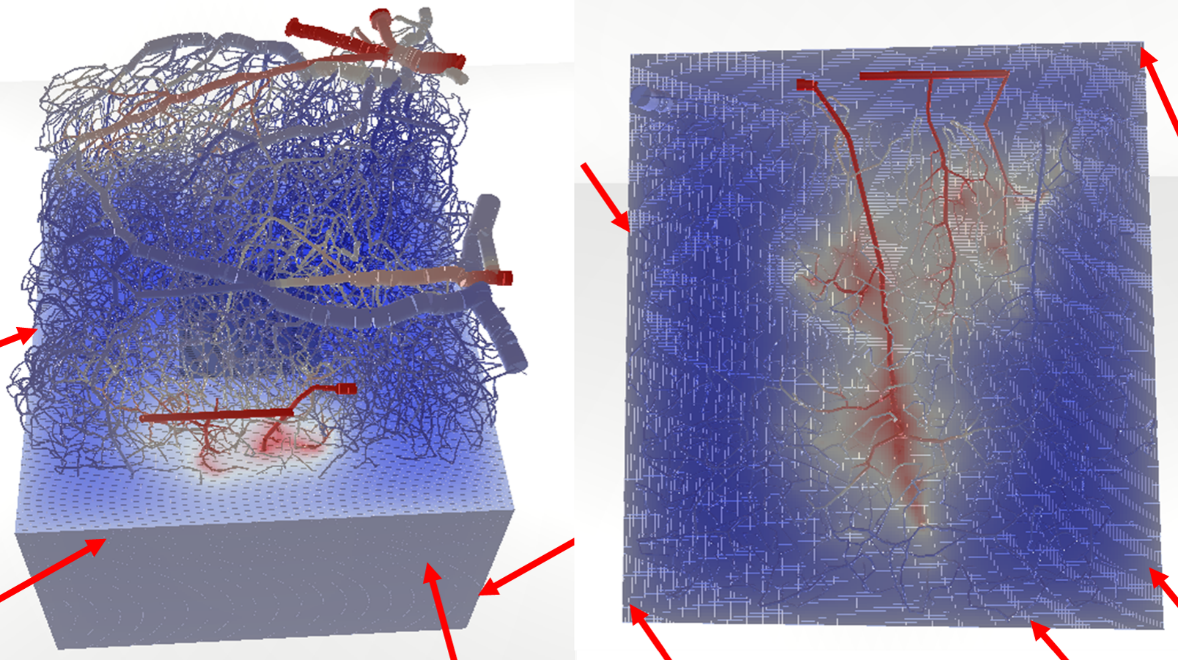 |
| --- |
| S4. Fig 1. Simulations of smaller networks have significant boundary effects (identified with red arrows) permeating throughout the simulation domain. This is not only due to the small size of the network, but is a limitation to the simulation domain; where the artificial boundary edges are not representative of the living tissue which has no such edges. The regions near the boundary then become devoid of vessels, which reduces blood flow and oxygen exchange to the surrounding tissue. To overcome these effects, in the main manuscript we successfully simulated a much larger domain, where the region of interest was far removed from the boundaries and thus did not observe these drastic effects. |
